# Reporting Conflicts of Interest and Funding in Healthcare Guidelines: The RIGHT-COI&F Checklist

**DOI:** 10.7326/M23-3274

**Published:** 2024-05-14

**Authors:** Yangqin Xun, Janne Estill, Joanne Khabsa, Ivan D. Florez, Gordon H. Guyatt, Susan L. Norris, Myeong Soo Lee, Akihiko Ozaki, Amir Qaseem, Holger J. Schünemann, Ruitai Shao, Yaolong Chen, Elie A. Akl, Author contributors of the RIGHT-COI&F Working Groups:, Yasser Sami Abdel Dayem Amer, Imad Bou Akl, Thurayya Arayssi, Pablo Alonso-Coello, Sarah Louise Barber, Stephanie Chang, Philipp Dahm, Yngve Falck-Ytter, Nathan Ford, Quinn Grundy, Glen S. Hazlewood, Akira Kuriyama, Andreas Lundh, Ana Marušić, Joseph L. Mathew, Barbara Mintzes, Reem Mustafa, David Fraile Navarro, Detty Nurdiati, Dawid Pieper, Hiroaki Saito, Yang Song, Renfeng Su, Rebekah Thomas, Ružica Tokalić, Marcello Tonelli, Ping Wang, Xu Wang, Zijun Wang, Nan Yang, Di Zhu

**Affiliations:** 1 School of Basic Medical Sciences, Lanzhou University, Gansu, China; 2 School of Population Medicine and Public Health, Chinese Academy of Medical Sciences & Peking Union Medical College, Beijing, China; 3 Institute of Global Health, University of Geneva, Geneva, Switzerland; 4 Clinical Research Institute, American University of Beirut Medical Center, Beirut, Lebanon; 5 Department of Pediatrics, University of Antioquia, Medellin, Colombia; 6 School of Rehabilitation Science, McMaster University, Hamilton, Ontario, Canada; 7 Pediatric Intensive Care Unit, Clínica Las Americas-AUNA, Medellin, Colombia; 8 Department of Health Research Methods, Evidence and Impact (HEI), McMaster University, Hamilton, Ontario, Canada; 9 Oregon Health & Science University, Portland, Oregon, USA; 10 Clinical Research Division, Korea Institute of Oriental Medicine, Daejeon, South Korea; 11 Department of Breast and Thyroid Surgery, Jyoban Hospital of Tokiwa Foundation, Iwaki City, Fukushima, Japan; 12 American College of Physicians, Philadelphia, Pennsylvania, USA; 13 Research Unit of Evidence Based Evaluation and Guidelines, Chinese Academy of Medical Sciences (2021RU017), Lanzhou University, Gansu, China; 14 WHO Collaborating Centre for Guideline Implementation and Knowledge Translation, Gansu, China; 15 Department of Internal Medicine, https://ror.org/04pznsd2 American University of Beirut, Beirut, Lebanon

## Abstract

**Background:**

Conflicts of interest (COI) of contributors to a guideline project and the funding of that project can influence the development of the guideline. Comprehensive reporting of information on COIs and funding is essential for the transparency and credibility of guidelines.

**Objective:**

To develop an extension of the RIGHT statement for the reporting of COIs and funding in policy documents of guideline organizations and in guidelines: the RIGHT-COI&F checklist.

**Design:**

The recommendations of the Enhancing the QUAlity and Transparency Of health Research (EQUATOR) network were followed. The process consisted of the following steps: 1) registration of the project and setting up working groups; 2) generation of the initial list of items; 3) achieving consensus on the items; and 4) formulating and testing the final checklist.

**Setting:**

International collaboration.

**Participants:**

44 experts.

**Measurements:**

Consensus on checklist items.

**Results:**

The checklist contains 27 items: 18 about the COIs of contributors and nine about the funding of the guideline project. Of the 27 items, 16 are labelled as policy-related as they address the reporting of COI and funding policies that apply across an organization’s guideline projects. These items should be described ideally in the organizations’ policy documents, otherwise in the specific guideline. The remaining 11 items are labelled as implementation-related and they address the reporting of COI and funding of the specific guideline.

**Limitations:**

The RIGHT-COI&F checklist requires testing in real-life use.

**Conclusion:**

The RIGHT-COI&F checklist can be used to guide the reporting of COIs and funding in guideline development, and to assess the completeness of reporting in published guidelines and policy documents.

**Primary Funding Source:**

The Fundamental Research Funds for the Central Universities of China.

## Introduction

Guidelines are “systematically developed evidence-based statements which assist providers, recipients, and other stakeholders to make informed decisions about appropriate health interventions”[1]. A conflict of interest (COI) is defined to exist when a past, current, or expected interest (which can be of financial, but also, for example, of intellectual or personal nature) creates a significant risk of inappropriately influencing an individual’s judgment, decision, or action when carrying out a specific duty[2]. COIs are common in guidelines[3, 4] and affect different steps of guideline development[5–7].

The development of high-quality guidelines requires not only methodological rigor[8], but also transparent and standardized reporting of the processes and content[9]. However, at present, the description of COIs and funding in guidelines tends to be poor: essential details are often lacking or the information is inconsistent[10–14], which in turn can seriously affect the transparency and threaten the credibility of the guideline.

Several reporting checklists such as the Conference on Guideline Standardization (COGS) checklist (2003)[15], the Appraisal of Guidelines for REsearch and Evaluation (AGREE) Reporting Checklist (2016)[16], and the Reporting Items for Practice Guidelines in healthcare (RIGHT) statement (2017)[9] have been developed to standardize the format and content of guidelines and improve the transparency of the entire development process. However, these comprehensive checklists have limited space to address specific topics, and COIs and funding are only covered superficially. At the same time, the influence of COI and funding remains very critical issue in guideline development that requires to be clearly and completely reported. There is thus a need for more detailed guidance on how to exactly report COIs and funding..

Aside from the limitations of existing reporting checklists, not all guideline development organizations have policies for COIs and funding[20, 21], and existing policies may not be publicly available. Moreover, guideline development requires multiple decisions based on the evidence, context, and values, and may have widespread impact on clinical practices and policies. Thus, funders or guideline developers’ relationships with external entities with an interest in the outcome of the process may have even greater influence on guidelines than on other types of research[22]. Guidelines are therefore particularly sensitive to influence caused by COIs or funders.

Therefore, we aimed to develop an extension of the RIGHT statement for the reporting of COIs and funding in policy documents of guideline organizations as well as in individual guidelines: the RIGHT-COI&F checklist.

## Methods

We developed the RIGHT-COI&F checklist in accordance with the guidelines for reporting health research[23] recommended by the Enhancing the QUAlity and Transparency Of health Research (EQUATOR) network, taking advantage of our experience from the development of the main RIGHT statement[9] and its published extensions[24–26].

**Figure 1:**
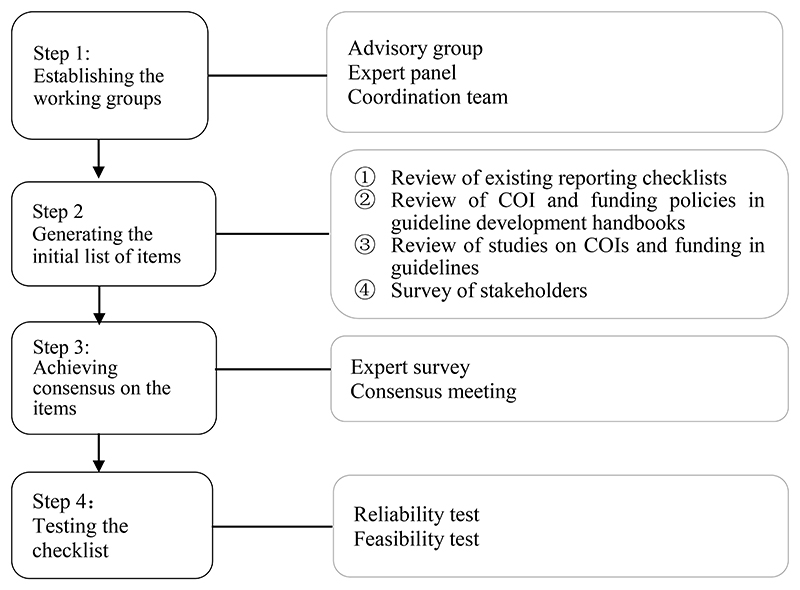
The four steps of developing the RIGHT-COI&F checklist. COI, conflict(s) of interest

We registered RIGHT-COI&F in the EQUATOR (https://www.equator—network.org/) collaboration network on July 15, 2021[27], and published a detailed protocol[28]. We illustrate the four steps of the development process in Figure 1 and describe them in detail below.

### 1 Establishment of the working groups

We formed three working groups: a coordination team, an advisory group, and an expert panel. We aimed to have a balanced representation of members with experience in guideline methodology, reporting checklist development, and COI research in all three groups. The coordination team first established the advisory group and both groups then together invited experts to the expert panel. All members of the working groups were required to declare their interests using a standardized form. The declarations were collected and evaluated by the coordination team.

The advisory group participated in the top-level design of RIGHT-COI&F, assisted in inviting participants to the expert panel, participated in the consensus meeting, reviewed and provided opinions and suggestions in different steps of the development, and reviewed the final checklist before publication.

The coordination team invited participants to the expert panel by contacting individuals who fulfilled at least one of the following conditions: experience in guideline methodology; experience in development of reporting checklists; or experience in research related to the influence of COI and/or funding. Members of the advisory group were requested to suggest experts with relevant experience. In addition, we extracted senior authors of selected relevant studies on the topic identified through a rapid search. We aimed for a balanced representation in terms of geographical location, gender, and specialty. The search was stopped after at least 20 individuals had accepted our invitation. The panel participated in an expert survey and consensus meeting and reviewed the final checklist.

The coordination team planned and conducted the entire process of the development of RIGHT-COI&F. The team generated the initial list of items; collected all participants’ interest disclosure forms and managed the COIs; prepared all documents and material; organized the meetings and surveys; collected and managed feedback; and drafted the final checklist. In addition, the coordination team supported and coordinated the work of the advisory group and the expert panel.

### 2 Generation of the initial list of items

We conducted a series of original studies to generate an initial list of items: 1) a review of existing reporting checklists on COI and funding related items[9, 15, 16]; 2) two reviews of COI and funding policies in guideline development handbooks[29, 30]; 3) a review of studies on COIs and funding in guidelines[31]; and 4) a questionnaire survey among guideline stakeholders to understand their knowledge on the topic and the needs for a reporting checklist on COIs and funding[32]. Two members of the coordination team collected all items identified in the studies independently, and then revised, merged and removed duplicates to form the initial pool of items. Next, the coordination team discussed, revised and condensed the content of the pool of items until consensus was reached. Afterwards, the list was sent to the Advisory group for feedback and revised accordingly to form the initial version of the RIGHT-COI&F checklist.

### 3 Achieving consensus on the items to include

#### 3.1 Expert survey

The coordination team used the Zoho system (https://www.zoho.com.cn/survey/) to conduct the survey. In the survey, the participants indicated their level of agreement with the inclusion of each item, using a 7-point Likert scale (1=strongly disagree, 7=strongly agree)[33]. Consensus was defined according to the study protocol [28]: items with a median score of at least 6 without any substantial comments were kept as such; items with a median score of 3 or below were excluded without further evaluation; otherwise, the item was revised according to the feedback. After reviewing the results of the survey, the coordination team replied to the comments given by the experts and proposed a revision of the checklist.

#### 3.2 Consensus meeting

All members of the working groups were invited to participate in the consensus meeting. Multiple consensus meetings were planned to ensure that as many working group members as possible could participate in the consensus process. The meetings were held online via Zoom (https://unige.zoom.us/). We emailed all relevant information and documents to the participants in advance.

The structure of the consensus meeting included an introduction presenting a summary of the expert survey, a moderated discussion on selected topics on the checklist’s structure and items, and a brief conclusion with suggested modifications to the checklist based on the discussion. The meetings were chaired by members of the coordination team who proposed topics for discussion based on the feedback given during the expert survey. Each topic was discussed until the participants found a proposed approach that received no objections. The coordination team recorded the consensus meetings and shared them with working group members who were unable to participate and asked for feedback. Subsequently, members of the coordination team reviewed all feedback, revised the checklist accordingly, and sent the final version to all members of the working groups for final approval.

### 4 Role of the funding source

This study was supported by the Fundamental Research Funds for the Central Universities (lzujbky-2021-ey13). The funders set no restrictions on how to use funding. The funders had no role in the study design, data collection and analysis, interpretation of data and writing of the article, or decisions to submit it for publication. M.S. Lee was supported by the Korea Institute of Oriental Medicine (KSN1823211). A. Marušić was funded by the Croatian Science Foundation (IP-2019-04-4882).

## Results

### 1 Basic information of the working groups

A total of 44 experts from 17 countries (Supplement 1 Figure 1) participated in the three working groups: five in the advisory group, 27 in the expert panel, and 12 in the coordination team (Supplement 1 Text 1). Eighteen (41%) participants were women. Twenty-two (50%) members had experience in guideline methodology, 13 (30%) in COI research, eight (18%) in health research reporting guidelines, 14 (32%) in clinical medicine, eight (18%) in public health, three (7%) in health statistics, and three (7%) in medical journal editing (Table 1). A minority of the working group members declared interests, such as receiving funding or fees not directly related to the present project, as well as participating in COI related research and activities, developing other reporting checklists, and serving in panels or boards related to COI management and research. After a review of the declarations, none of the reported interests were deemed to constitute COI and no participant was excluded from any step of the development.

### 2 Formulating the RIGHT-COI&F checklist

Supplement 1 Figure 2 shows the process of selecting items for the RIGHT-COI&F checklist. The four original studies generated a pool of 42 items, which was used to produce an initial checklist of 32 items.

We conducted the expert survey between December 17, 2022 and January 17, 2023. All 27 experts completed the survey. The median scores of the items ranged between 5 and 7 in the first round. Twenty-seven of the 32 items had a median score of at least 6, and all remaining five items had a median score of 5. In addition, a total of 177 free-text comments were given by the experts. Of the 27 items with a median score of 6 or above, eight were kept unaltered, 16 were revised based on the free-text comments, and five were removed because their content was already covered by other items after the revisions. According to the protocol, the five items with a median score of 5 should have been taken to the second round. However, after investigating the comments received on these items in detail, we found that the suggestions of the experts either exceeded the scope of the checklist or were already covered by other items. Based on a careful review of these comments, we decided to retain two of these five items, drop the remaining three, omit the second round of the expert survey, and proceed directly to the consensus meetings. Supplement 2 presents the full results of the survey including the revisions and changes made to the items.

We organized three separate consensus meetings on February 22, 23, and 27, 2023, to accommodate the large number of experts and their different time zones. Thirty participants from all working groups attended at least one meeting. The discussions led to the refinement of the number of items in the checklist, a better conceptualization of the relationship of the checklist with the original RIGHT statement, and changes in the order of items, terminology, and item wording. Based on the consensus meetings, the coordination team made revisions to eight items. Supplement 3 presents a summary of the key comments given during the meetings with corresponding responses.

### 3 The final RIGHT-COI&F Checklist

Table 2 provides the final RIGHT-COI&F checklist. Supplement 4 provides a detailed explanation and elaboration of each checklist item while Table 3 presents a glossary of the main terms and concepts used in the checklist. The checklist consists of 27 items organized in nine topics under two sections. The section on the COIs of guideline contributors consists of 18 items grouped into six topics. The section on the funding of the guideline project itself consists of nine items organized into three topics.

Concurrently, each checklist item is labeled as either policy-related (n=16), or implementation-related (n=11). The policy-related items describe how the principles of COI and funding management should be reported, for example, what types of interests need to be declared, and how these interests should be assessed. These items would typically apply to all guideline projects produced by the organization. The implementation-related items apply to the specific guideline project that is being reported on, covering, for example, the actual declarations by the panel members and the funding received by the guideline. Therefore, authors of guidelines may refer to the organizational policy (typically in the form of a guideline handbook) for any of the policy-related items that are covered there and report on the remaining items (including the eight implementation-related items) in the guideline report. This means also that the number of applicable items in most individual guideline projects is likely to be substantially less than 27, which further expedites the use of the RIGHT-COI&F checklist.

If the organizational policy however does not properly adhere to all policy-related items of RIGHT-COI&F, the content of these items should be reported in the guideline itself. If the developer organization does not have a COI policy, the entire checklist should be used when developing the guideline (Supplement 1 Figure 3).

## Discussion

RIGHT-COI&F is the first checklist specifically designed for reporting COIs and funding in guideline development organizations’ policy documents and in individual guidelines. This checklist provides detailed guidance on how to report information related to COIs and funding, which will enhance the completeness of this information and promote dissemination and implementation of guidelines[23]. The checklist is applicable to any type of guideline, regardless of target population or health care setting. At the same time, RIGHT-COI&F complements rather than replaces the current COI- and funding-related items in the main checklist of the RIGHT Statement (items 18a, 18b, 19a, 19b)[9]. Any guideline should adhere to the main RIGHT Statement, including these four items. RIGHT-COI&F serves guideline developers seeking guidance for reporting COIs and funding. Researchers can also use the RIGHT-COI&F checklist to assess the quality of the reporting of contents related to COIs and funding in guidelines and policy documents.

The purpose of this reporting checklist is not to provide instructions on how to declare and manage the interests and funding, but to guide the reporting of the content. Comprehensive reporting is the fundament of transparent dissemination of research findings[23]. When all essential information is reported and easy to find, guideline users can easily judge whether and to what extent the COIs and funding influence the recommendations of the guideline. Although we recommend that authors report all content in accordance with RIGHT-COI&F within the text or annexes of the guidelines or policies, we have kept it flexible for authors to choose where, in which order, and in which format the content is reported. The order of items is based on the general steps in the process of guideline development[2].

Considering the length of the checklist, having too many items may be counterproductive. We therefore attempted to keep the number of items as low as possible while still covering all key aspects related to COIs and funding. Individual guidelines need to follow only the implementation-related items if their organizational policy adheres to the checklist. Therefore, we anticipate that for most guidelines the length of the applicable checklist is feasible, despite the relatively high total number of items in RIGHT-COI&F.

The reporting checklist strictly distinguishes between COIs of individual contributors and the funding of the guideline project. Although COIs and funding may both bias guidelines, the associated risks are assessed differently. For COI, one would evaluate the relevance, nature, magnitude, and recency of the declared interests[2]. For funding, one would primarily evaluate the degree of involvement of the funder in the development of the guidelines.

This study has several strengths. First, RIGHT-COI&F adopted an internationally recognized procedure for developing reporting guidelines, which ensures the rigor of the development methods. Second, the project was registered on the EQUATOR platform, and the study protocol was published at the same time, which enhances the transparency of the process. Third, the members of the working groups represented a broad range of fields and geographical settings, and most members had rich working experience, authority and influence in the fields of evidence-based medicine, guideline methodology, COI research, and reporting guidelines.

The study also has limitations. First, we used an expert survey followed by consensus meetings to collect feedback from experts and reach consensus on the final format of the checklist. Although this approach is recommended for use in developing health research reporting guidelines[23], the method also has limitations. For example, in the consensus meetings the experts may adjust their opinions to align better with those of the group or the facilitators. Second, the guideline stakeholder survey was only carried out in selected countries in Asia, which may affect the representativeness of the survey results. The survey is however only one of several sources that contributed to the initial pool of items. The other three sources (a review of existing reporting guidelines, two reviews of guideline COI and funding policies, and a cross-sectional survey of their current state of studies on COIs and funding in guidelines) had global coverage, and the consensus experts came from 17 countries on five continents. Third, the RIGHT-COI&F checklist requires further testing in real-world use. We will test the feasibility and reliability of the checklist in a separate study by applying the checklist to existing guidelines and policy documents and conducting a survey among target users. Finally, although we asked all working group members to declare their interests, we cannot completely exclude the possibility of conflicts as the disclosures were based on self-reporting.

To promote the dissemination and implementation of the RIGHT-COI&F checklist and increase its influence, the coordination team members will disseminate the checklist through their contact networks and in academic conferences. We will also translate the reporting checklist into multiple languages and make it freely available on the RIGHT and EQUATOR websites. We will actively promote the checklist for endorsement by medical journals publishing guidelines. We plan also to actively collect new evidence and user feedback related to the content of the items to update the checklist, as well as regularly assess changes in the reporting of COIs and funding in guidelines to estimate the impact of the RIGHT-COI&F checklist.

## Conclusion

RIGHT-COI&F is a comprehensive checklist that provides guidance on how to report information on COIs and funding in guidelines and guideline policy documents. The checklist was developed by a multidisciplinary international team of experts in strict accordance with the development method of guidelines for reporting health research. The items were collected through a systematic search of evidence, an expert survey and consensus meetings. By guiding and standardizing the writing and presentation of information on COIs and funding in guidelines and guideline development organizations’ policies, RIGHT-COI&F can improve the transparency of guidelines, and ultimately minimize the risks caused by COIs and funding in healthcare.

## Supplementary Material

Supplemental file 1

Supplemental file 2

Supplemental file 3

Supplemental file 4

## Figures and Tables

**Table 1:** Characteristics of the members of RIGHT-COI&F working groups

Characteristic	Coordination Team(n=12)	Advisory Group(n=5)	Expert Panel(n=27)	Total(n=44)
**Continent, n (%)**				
Asia	10 (83)	-	11 (41)	21 (48)
Oceania	-	-	2 (7)	2 (5)
Europe	2 (17)	-	7 (26)	9 (21)
North America	-	4 (80)	7 (26)	11 (25)
South America	-	1 (20)	-	1 (2)
**Country income level, n (%)**				
High-income	2 (17)	4 (80)	23 (85)	29 (66)
Upper-middle income	8 (67)	1 (20)	2 (7)	11 (25)
Lower-middle income	2 (17)	-	2 (7)	4 (9)
**Field of expertise, n (%)**				
Clinical medicine	2 (17)	1 (20)	11 (41)	14 (32)
Conflict of interest research	2 (17)	2 (40)	9 (33)	13 (30)
Evidence-based medicine	5 (42)	2 (40)	8 (30)	15 (34)
Guideline methodology	7 (58)	3 (60)	12 (44)	22 (50)
Health statistics	3 (25)	-	-	3 (7)
Journal editor	-	-	2 (7)	2 (5)
Public health	3 (25)	1 (20)	4 (15)	8 (18)
Reporting guidelines	4 (33)	1 (20)	3 (11)	8 (18)
Other	-	-	1 (4)	1 (2)

**Table 2:** The RIGHT-COI&F checklist

Section/Topic	No.	Policy-related items (organization specific)	Implementation-related items (guideline project specific)	Page	Notes
**Conflicts of interest (COI) of contributors to the guideline project**					
Public access to the information	1		Indicate which COI policy was implemented (e.g., the organization’s COI policy, policy developed specifically for the guidelines), and how to access it.		
Definitions	2	State the definition and categorization of COI used by the guideline development organization.			
Preparations for COI management	3	State who is responsible for implementing the organization’s COI policy, (e.g., a committee independent of the guideline development group), and, if applicable, describe the details (e.g., the establishment process, composition, whether standing or ad hoc committee)			
	4	Describe the actions applied prior to the formation of the guideline development group to minimize COI (e.g., screening publicly available DOI/COI databases, inviting only contributors with no COI).			
Declaration of interests	5	Describe to which groups contributing to the guideline project the policy applies (e.g., guideline development group, systematic reviewers, peer reviewers).			
	6	Describe whether the individuals declaring their interests should also declare the interests of other individuals related to them and specify who those individuals are (e.g., spouse).			
	7	Describe in which format the interests should be declared (e.g., whether a standardized form was used).			
	8	Describe what interests should be declared (e.g., according to type of interest, relevance to the topic, the source of the interest, a minimum amount for financial interest, or the recency).			
	9	Describe what details of the interests (e.g., source, amount, date) should be declared.			
	10	Describe any process used for updating declarations of interests (e.g., frequency, schedule, format, procedure to remind/collect the updated interests).			
	11		Report the declarations of interests or a comprehensive summary of them (initial ones and any updates), including declarations of ‘no interest’.		
Assessment of interests	12	Describe any process used to verify the accuracy and completeness of declarations (e.g., responsible individual, method of verification, how discrepancies between sources are dealt with).			
	13	Describe the criteria used for assessing whether an interest qualifies as a COI and any assessment of the level of risk associated with the COI.			
	14		Report the results of the assessment of whether the declared interests were considered COI.		
Management of COI	15	Describe the COI management strategy and how (if applicable) it accounts for the level of the risk associated with the COI [e.g., requiring a minimum percentage of panelists free from COI, exclusion from the panel, exclusion from specific roles (e.g., chair, systematic reviewer), exclusion from specific aspects for the process (e.g., voting), divestment, restriction from relations that could lead to COI during/after assignment]			
	16	Describe any implications for non-compliance with rules of declaration.			
	17	Describe any process to resolve disputes in the implementation of the COI policy.			
	18		Report the results of the COI management strategy (e.g., whether individuals were excluded or their contribution was restricted)		
**Funding of the guideline project**					
Public access to the information	19		Indicate which funding policy was implemented (e.g., the organization’s funding policy, policy developed specifically for the guidelines), and how to access it.		
The source of funding	20	Indicate whether funding should not be accepted from specific sources, if applicable.			
	21	Indicate whether the amount of funding should be reported.			
	22		Report whether the guideline received or is expected to receive funding, whether direct or indirect (if not, items 23-25 are not applicable)		
	23		Provide the name(s) of the direct or indirect funder(s)		
	24		Provide the identifiers for the funding (e.g., grant number), if applicable.		
	25		Indicate whether the funder(s) set any restrictions on how to use the funding.		
	26		Describe the role of funder(s) in the different steps of guideline development, planned dissemination and planned implementation.		
Management of the funding	27		Describe any mitigation strategies (e.g., use of a funding firewall) to minimize the influence of the funder(s) on the guideline development process.		

**Table 3:** Glossary of terms for the RIGHT-COI&F checklist

Term	Definition
Practice guideline	A statement that includes recommendations intended to optimize patient care that are informed by a systematic review of evidence and an assessment of the benefits and harms of alternative care options[34].
Conflict of interest (COI)	A COI exists when a past, current, or expected interest creates a significant risk of inappropriately influencing an individual’s judgment, decision, or action when carrying out a specific duty[2].
Interest	A benefit (e.g., money received from the industry) or an attribute of the individual (e.g., having specific religious beliefs). The existence of an interest does not necessarily imply the existence of a conflict of interest[2, 35, 36].
Guideline development policy	A set of internal regulations and instructions for the development of the guidelines. The policy is usually described in the developer guideline manual.
Disclosure of interests (DOI)	Listing of all interests that may lead to conflicts of interests. All declared interests should be evaluated for whether they constitute a COI.
Funding of guideline	Money or resources provided to the guideline project itself to support its development, dissemination and implementation, and any other related activities.
Direct funding	Funding of a guideline project that is explicitly declared as such.
Indirect funding	Funding of a guideline project that is not explicitly declared as such, but is actually used to support the direct funders of the guideline project.
