# Supplementary material for: Reporting Conflicts of Interest and Funding in Healthcare Guidelines: The RIGHT-COI&F Checklist
Source: Ann Intern Med. Author manuscript; Available in PMC 2024 Dec 24. (PMC7616250; doi:10.7326/M23-3274)
Supplement: Supplemental file 1 [file EMS196956-supplement-Supplemental_file_1.docx]

**Supplement 1: Supplementary figures and the list of working group members**


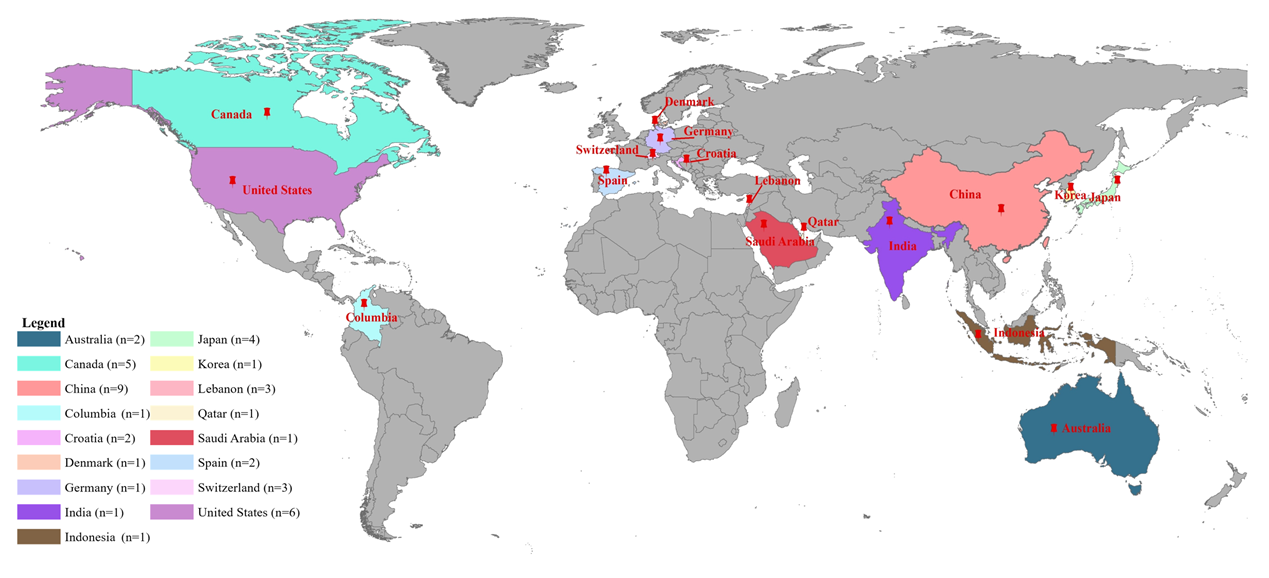


**Supplement 1 Figure 1. Countries of the members of the RIGHT-COI&F working groups**

Items merged and removed

Revised: 12 items

Unchanged: 18 items

New items added: 2 items

Removed: 8 items

Revised: 16 items

Unchanged: 8 items

New items added: 3 items

Drafting by Coordination team

Pool of initial items

(N=42)

Removed: 12 items

Revised: 10 items

Unchanged: 9 items

Merged: 4 into 1

New items added: 10 items

35 items

30 items

32 items

27 items

27 items

Multiple discussions among the Coordination team

Suggestions from Advisory group

Expert survey

Consensus meeting

Revised: 11 items

Unchanged: 16 items

**Supplement 1 Figure 2**. **Selection of the items for the RIGHT-COI&F checklist**

Implication for **guideline reports**

**AND**

Policy items only

(16 items)

Implication for **guideline policy documents**

Does the guideline report refer to a publicly available policy document?

**Yes**

**No**

Policy items not reported in the policy document (0 to 16 items)

All policy items

(16 items)

All implementation items

(11 items)

**OR**

**Supplement 1 Figure 3. A diagram on which items of the RIGHT-COI&F checklist are applicable in different situations.** For guideline policy documents, only the policy items are applicable. For guideline reports, all implementation items and the policy items that are not reported in a publicly available, referenced policy document are applicable.

**Supplement 1 Text 1. List of members of RIGHT-COI&F working groups**

**RIGHT-COI&F Coordination Team (in alphabetical order)**

- Elie Akl. Department of Internal Medicine, American University of Beirut, Beirut, Lebanon; Department of Health Research Methods, Evidence, and Impact (HEI), McMaster University, Hamilton, ON, Canada.
- Yaolong Chen. School of Basic Medical Sciences, Lanzhou University, Lanzhou, China.
- Janne Estill. Institute of Global Health, University of Geneva, Geneva, Switzerland; School of Basic Medical Sciences, Lanzhou University, Lanzhou, China.
- Joanne Khabsa. Clinical Research Institute, American University of Beirut Medical Center, Beirut, Lebanon.
- Yang Song. Iberoamerican Cochrane Center - Biomedical Research Institute Sant Pau (IIB Sant Pau), Barcelona, Spain.
- Renfeng Su. School of Public Health, Lanzhou University, Lanzhou, China.
- Yangqin Xun. School of Basic Medical Sciences, Lanzhou University, Lanzhou, China; School of Population Medicine and Public Health, Chinese Academy of Medical Sciences & Peking Union Medical College, Beijing, China.
- Ping Wang. School of Basic Medical Sciences, Lanzhou University, Lanzhou, China.
- Xu Wang. Chevidence Lab of Child and Adolescent Health, Children's Hospital of Chongqing Medical University, Chongqing, China.
- Zijun Wang. Evidence-Based Medicine Center, School of Basic Medical Sciences, Lanzhou University, Lanzhou, China; Institute of Global Health, University of Geneva, Geneva, Switzerland.
- Nan Yang. Evidence-Based Medicine Center, School of Basic Medical Sciences, Lanzhou University, Lanzhou, China.
- Di Zhu. School of Public Health, Lanzhou University, Lanzhou, China.

**RIGHT-COI&F Advisory Group (in alphabetical order)**

- Ivan D. Florez. Department of Pediatrics, University of Antioquia, Medellin, Colombia; School of Rehabilitation Science, McMaster University, Hamilton, Ontario, Canada; Pediatric Intensive Care Unit, Clínica Las Americas-AUNA, Medellin, Colombia.
- Gordon Guyatt. Department of Health Research Methods, Evidence and Impact, McMaster University, ON, Canada.
- Susan L. Norris. Oregon Health & Science University, Portland, Oregon, USA.
- Holger J. Schünemann. Department of Health Research Methods, Evidence, and Impact (HEI), McMaster University, Hamilton, Canada.
- Amir Qaseem. American College of Physicians, Philadelphia, Pennsylvania, USA.

**RIGHT-COI&F Expert Panel (in alphabetical order)**

- Yasser Sami Abdel Dayem Amer. Pediatrics Department and Clinical Practice Guidelines & Quality Research Unit, Corporate Quality Management Department, King Saud University Medical City, Riyadh, Saudi Arabia.; Research Chair for Evidence-Based Health Care and Knowledge Translation, King Saud University, Riyadh, Saudi Arabia; Alexandria Center for Evidence-Based Clinical Practice Guidelines, Alexandria University, Egypt; Department of Internal Medicine, Ribeirão Preto Medical School, University of São Paulo (FMRP-USP), Brazil.
- Imad Bou Akl. Department of Internal Medicine, American University of Beirut, Lebanon, Lebanon.
- Thurayya Arayssi. Weill Cornell Medicine-Qatar, Doha, Qatar.
- Pablo Alonso-Coello. Iberoamerican Cochrane Center - Biomedical Research Institute Sant Pau (IIB Sant Pau), Barcelona, Spain. CIBER de Epidemiología y Salud Pública (CIBERESP), Barcelona, Spain.
- Sarah Louise Barber. Centre for Health Development, World Health Organization, Kobe, Japan.
- Stephanie Chang. Deputy Editor, Annals of Internal Medicine, and American College of Physicians, Washington, DC.
- Philipp Dahm. Urology Section, Minneapolis VAMC and Department of Urology, University of Minnesota, Minneapolis, Minnesota, USA.
- Yngve Falck-Ytter. Department of Veterans Affairs Cleveland Medical Center, Cleveland, Ohio; Case Western Reserve University, Cleveland, Ohio.
- Nathan Ford. Department of Global HIV, Hepatitis and Sexually Transmitted Infections Programmes, World Health Organization, Geneva, Switzerland.
- Quinn Grundy. Lawrence Bloomberg Faculty of Nursing, University of Toronto, Toronto, ON, Canada.
- Glen S. Hazlewood. Department of Medicine, Cumming School of Medicine, Calgary, Canada.
- Akira Kuriyama. Department of Primary Care and Emergency Medicine, Kyoto University Graduate School of Medicine, Kyoto, Japan.
- Myeong Soo Lee. Science Research Division, Korea Institute of Oriental Medicine, Daejeon, South Korea.
- Andreas Lundh. Cochrane Denmark & Centre for Evidence-Based Medicine Odense, Department of Clinical Research, University of Southern Denmark, Denmark; Department of Respiratory Medicine and Infectious Diseases, Copenhagen University Hospital - Bispebjerg and Frederiksberg, Denmark.
- Ana Marušić. Department of Research in Biomedicine and Health, Center for Evidence-based Medicine, University of Split, Split, Croatia.
- Joseph L. Mathew. Advanced Pediatrics Centre, PGIMER, Chandigarh, India; Advanced Centre for Evidence Based Child Health (ACEBCH), Advanced Pediatrics Centre, Postgraduate Institute of Medical Education and Research (PGIMER), Chandigarh, India.
- Barbara Mintzes. School of Pharmacy and Charles Perkins Centre, Faculty of Medicine and Health, University of Sydney, Sydney, NSW, Australia.
- Reem Mustafa. Department of Health Research Methods, Evidence and Impact, McMaster University, Hamilton, Ontario, Canada.
- David Fraile Navarro. Australian Living Evidence Collaboration (ALEC), Cochrane Australia, School of Public Health and Preventive Medicine, Monash University, Melbourne, Australia; Australian Institute of Health Innovation, Macquarie University, Sydney, Australia.
- Detty Nurdiati. Clinical Epidemiology and Biostatistics Unit, Faculty of Medicine, Public Health and Nursing, Universitas Gadjah Mada/Dr. Sardjito General Hospital, Yogyakarta, Indonesia.
- Akihiko Ozaki. Department of Breast and Thyroid Surgery, Jyoban Hospital of Tokiwa Foundation, Iwaki City, Fukushima, Japan.
- Dawid Pieper. Faculty of Health Sciences Brandenburg, Brandenburg Medical School (Theodor Fontane), Institute for Health Services and Health Systems Research, Rüdersdorf, Germany; Center for Health Services Research, Brandenburg Medical School (Theodor Fontane), Rüdersdorf, Germany.
- Hiroaki Saito. Department of Internal Medicine, Soma Central Hospital, Fukushima, Japan; Medical Governance Research Institute, Tokyo, Japan.
- Ruitai Shao. School of Population Medicine and Public Health, Chinese Academy of Medical Sciences & Peking Union Medical College, Beijing, China.
- Rebekah Thomas. Guidelines Review Committee, Methods and Standards Unit, Science Division, World Health Organization, Geneva, Switzerland.
- Ružica Tokalić. Department of hematology, Clinical Hospital Holy Spirit, Zagreb, Croatia.
- Marcello Tonelli. Department of Medicine, University of Calgary, Calgary, Alberta, Canada.
