# Supplementary material for: Reporting Conflicts of Interest and Funding in Healthcare Guidelines: The RIGHT-COI&F Checklist
Source: Ann Intern Med. Author manuscript; Available in PMC 2024 Dec 24. (PMC7616250; doi:10.7326/M23-3274)
Supplement: Supplemental file 2 [file EMS196956-supplement-Supplemental_file_2.docx]

**Supplement 4. The extension of the RIGHT Statement for reporting conflicts of interest and funding in practice guidelines: explanation and elaboration**

1. **Background**

Reporting checklists often include technical terms that may be challenging for readers to understand[1]. An explanation and elaboration document can help clarify the guidance using simple, accurate and clear language. This would in turn increase compliance with the guidance. In addition, the explanation and elaboration document can also provide examples and suggestions to help readers better apply the items.

The Reporting Items for practice Guidelines in HealThcare (RIGHT statement) was published in the Annals of Internal Medicine in 2017 together with an explanation and elaboration document[2].

The present document contains the explanation and elaboration materials for the extension of RIGHT for conflicts of interest (COI) and funding, the RIGHT-COI&F reporting checklist. This will help the users better understand and apply RIGHT-COI&F, and ensure a standardized reporting of COI and funding in practice guidelines as well as in organizational policies for guideline development. The items marked with an asterisk (*) are implementation-related, i.e. they address the implementation of COI and funding policies in individual guideline reports. The remaining items are policy-related, i.e. they address the COI and funding policies of different organizations which may be reported either in the organizational policy documents or the guidelines themselves.

1. **The items of the RIGHT-COI&F checklist with explanations and examples**

**Section 1:** **Conflicts of interest (COI) of contributors to the guideline project**

**Item 1*:** Indicate which COI policy was implemented (e.g., the organization’s COI policy, policy developed specifically for the guidelines), and how to access it.

**Explanation:** The guideline report should indicate whether the guideline was developed in accordance with an existing COI policy (either the developer organization’s own policy or another published policy) or a COI policy was developed specifically for this guideline. The report should also indicate how to access the policy (e.g., link, full bibliographic details). This is not only to ensure the transparency of the declaration and management of the interests of this guideline, but also to serve as a reference for future guideline developers and COI researchers. Different COI policies require interests to be declared and managed in different ways and formats, and a clear reference to the implemented COI policy will help readers judge the guidelines’ adherence to the COI policy and enhance the reliability of the guideline.

**Example:** *“Guidelines for declaration of interests (WHO experts) are available at: https://www.who.int/about/ethics/doi-guide-EN.pdf?ua=1.” [3]*

**Item 2:** State the definition and categorization of COI used by the guideline development organization.

**Explanation:** Although some organizations have published definitions and categorizations for COI, there is no unified definition. A clear definition of COI, as well as a categorization of the COI (e.g., financial vs non-financial; individual vs. institutional) can ensure a shared understanding of COI by guideline contributors who declare, assess or manage them.

**Examples:** *“[COI is defined as] any interest declared by an expert that may affect or reasonably be perceived to affect the expert’s objectivity and independence in providing advice to WHO.”[4]*

*“Conflicts of interest are of two basic types: financial and nonfinancial. Both are relevant to guideline development. Financial interests can be directly measured in monetary units, such as for stocks or patents owned, money received for commissioned work, or an honorarium for a speaking engagement. Nonfinancial interests, on the other hand, cannot readily be measured in monetary units and are less tangible and thus more difficult to identify, measure and manage. They include any interest that could be reasonably perceived to affect an individual’s objectivity and independence while working with WHO.”[4]*

**Item 3:** State who is responsible for implementing the organization’s COI policy, (e.g., a committee independent of the guideline development group), and, if applicable, describe the details (e.g., the establishment process, composition, whether standing or ad hoc committee)

**Explanation:** The COI policy is typically implemented and overseen by individuals or committees having no COI themselves. Some guideline development organizations may have a permanent COI management unit that is responsible for managing COIs in guideline development and other work of the organization. The establishment and recruitment of the entity responsible for the implementation of the COI policy will affect the management of COI, and clear reporting of details such as the establishment process, composition and nature of the committee will help to provide clearer guidance to the users of the policy, i.e. those involved in the development of the guideline. The entity may be, e.g., and individual, a group of individuals, an external ad hoc committee, or the organization’s COI management unit.

**Examples:** *“Disclosures for each potential member will be reviewed by staff and the chair of the GDG prior to placement on the panel.”[5]*

*“The guideline facilitation team, composed of an AAN* *(American Academy of Neurology) EBM (Evidence-Based Medicine) methodologist, AAN staff, and GDDI leadership, reviews each form before the prospective panel member is officially invited to begin work on the guideline project. The facilitation team reviews the relationship disclosures for any relevant relationships that may constitute a conflict of interest[6].*

*The Conflict of Interest Committee reviews disclosures by candidates for the CPG and GWGs before their appointment by the APA President and approval by the APA Board of Trustees.”[7]*

**Item 4:** Describe the actions applied prior to the formation of the guideline development group to minimize COI (e.g., screening publicly available DOI/COI databases, inviting only contributors with no COI).

**Explanation:** In order to reduce the COI of the members of the guideline working group, some guideline development organizations may check the interests and relationships of the proposed members before forming the guideline working group. This can be done by asking potential members to first declare their interest, or by searching their declared interests in publicly accessible DOI/COI databases. The decision to invite them to serve on the guideline working group can be made accordingly. In this way, COIs can be minimized before initiating the actual development process.

**Example:** *“The guideline facilitation team, composed of an AAN EBM methodologist, AAN staff, and GDDI (Guideline Development, Dissemination, and Implementation Subcommittee) leadership, reviews each form before the prospective panel member is officially invited to begin work on the guideline project. The facilitation team reviews the relationship disclosures for any relevant relationships that may constitute a conflict of interest.”[6]*

**Item 5:** Describe to which groups contributing to the guideline project the policy applies (e.g., guideline development group, systematic reviewers, peer reviewers).

**Explanation:** A guideline project may involve a large number of individuals with differing levels of contribution. Given that different guideline developers require some but not all contributors to declare their interests, it is necessary to clearly describe which contributors were required to do so.

**Example:** *“According to WHO rules and regulations, a declaration of interests form and an analysis of all declarations must be performed whenever an individual provides independent advice to WHO (7) − that is, whenever an expert is asked to serve in an advisory role in a personal capacity. In guideline development, this means that anyone invited to participate in the development of a WHO guideline must complete the declaration of interests form and agree to the publication of a summary of that declaration in the guideline (see Table 6.1 and Fig. 6.1). This includes all members of the GDG, individuals who prepare systematic reviews and evidence profiles, guideline methodologists, the technical writer and any other expert who participates in the process in an individual capacity.”[4]*

**Item 6:** Describe whether the individuals declaring their interests should also declare the interests of other individuals related to them and specify who those individuals are (e.g., spouse).

**Explanation:** Most COI guidance require contributors to declare the interests of closely related individuals, such as spouses and children. Such interests may create COI that influence the process of the guideline development.

**Examples:** *“Relevant financial as well as nonfinancial interests should be disclosed and subsequently assessed and managed in order to minimize bias in guideline development and to ensure credible recommendations. A financial conflict of interest arises when an individual or organization receives income or monetary support that is related to, or could be affected by, the outcome of the WHO meeting or activity in which they are involved. This includes both personal financial interests and the interests of the individual’s immediate family members (defined as the spouse, or partner with whom one has a close personal relationship, and the children).”[4]*

*“Information regarding the interests of a panel candidate’s spouse and dependents is gathered as part of the disclosure process. Candidates should report, to the best of their ability, any known interests of his/her immediate family that may be related to the guideline topic under consideration.”[8]*

**Item 7:** Describe in which format the interests should be declared (e.g., whether a standardized form was used).

**Explanation:** Interests are usually declared in a standardized format, i.e. members of the guideline working group are required to complete a declaration of interests form, which covers the scope, content and related details of the interests to be declared in a standardized manner. It is conducive to members declaring the relevant interests clearly and accurately.

**Example:** *“All prospective participants complete a DOI form before engaging in any CGC work. (…) Participants involved in the development of ACP guidelines disclose interests using a structured electronic format with specific categorical prompts.”[9]*

**Item 8:** Describe what interests should be declared (e.g., according to type of interest, relevance to the topic, the source of the interest, a minimum amount for financial interest, or the recency).

**Explanation:** COI policies need to clearly state what interests should be declared. Details that may be relevant to describe include whether the policy applies to non-financial interests in addition to financial interests; how relevant the interests need to be to the subject matter of the guideline; the types of entities the relationships with which need to be declared; any amount of financial benefits used as a threshold for declaration; and any time frame used to determine the interests that need to be declared. For example, the threshold for reporting financial relationships might depend on the local context and therefore may need to be clearly reported.

**Example:** *“Relevant financial as well as nonfinancial interests should be disclosed and subsequently assessed and managed in order to minimize bias in guideline development and to ensure credible recommendations. A financial conflict of interest arises when an individual or organization receives income or monetary support that is related to, or could be affected by, the outcome of the WHO meeting or activity in which they are involved. This includes both personal financial interests and the interests of the individual’s immediate family members (defined as the spouse, or partner with whom one has a close personal relationship, and the children). Financial interests include, for example:*

*■ personal financial gain such as paid work, consulting income or honoraria and travel stipends;*

*■ support for research, including direct monetary contributions or donations of equipment, laboratory space, etc.;*

*■ proprietary interests and patents;*

*■ grants, fellowships or other financial support to the individual or to their institution; and*

*■ stock shares or bonds in a commercial entity.*

*The threshold for reporting relevant personal investment interests in the WHO declaration of interests form is 5000 United States dollars (US$): when the value of a single interest is below that amount, it does not have to be reported. Research support must also be disclosed, including non-monetary support valued at more than 1000 US$.”[4]*

**Item 9:** Describe what details of the interests (e.g., source, amount, date) should be declared.

**Explanation:** While the previous item (8) addresses the criteria of when interests need to be reported, the present item lists the details of the interests (aligned with the criteria mentioned in the previous item) that need to be disclosed.

**Examples:** *“Disclosure of Financial Interests: Task Force Members.”[10]*

|  | Type of Financial Interest | Amount ($) | Description | Date Potential COI Occurred (mm/yy) |
| --- | --- | --- | --- | --- |
| Task Force Member Name |  |  |  |  |
| Task Force Member Name |  |  |  |  |

*“Where applicable, only the source of the income and the nature of the COI are to be disclosed; the amount of any payment or grant etc. is not required[11].”*

**Item 10:** Describe any process used for updating the declarations of interests (e.g., frequency, schedule, format, procedure to remind/collect the updated interests).

**Explanation:** Updating is one of the key steps in the declaration and management of interests. The original declaration of interest is usually submitted before or when the guideline contributor joins the working group. However, the development of a guideline takes generally 2-5 years[12], and the interest of the guideline contributor may change during this period. It may therefore be necessary to also update the declarations of interest. Guideline development organizations should describe the details of the process for updates such as the frequency, schedule, and form of the update statement.

**Example:** *“Panel members must update their Relationship Disclosure Form at least annually but also promptly at any time a relationship change.”[6]*

**Item 11*:** Report the declarations of interests or a comprehensive summary of them (initial ones and any updates), including declarations of ‘no interest’.

**Explanation:** Usually, all contributors to the development of the guideline are requested to declare their interests and disclose the information to the readers of the guideline. This includes both the initial declarations of interest before the guideline development process was initiated, as well as the updates during the guideline development process. Even if there are no interests that need to be declared, the lack of interests should be explicitly reported in the guideline. Considering that some guideline projects have a large number of participants, a comprehensive summary of the interests can be provided instead of the original declarations.

**Example:** *“WHO guidelines for the prevention of sexual transmission of Zika virus reported that: a review of the disclosure of interest form concluded that there was no COI (see Annex A1 in this guideline for details). Therefore, participation in the guideline development process did not exclude any member.”[13]*

**Item 12:** Describe any process used to verify the accuracy and completeness of declarations (e.g., responsible individual, method of verification, how discrepancies between sources are dealt with).

**Explanation:** Considering that underreporting of financial COI is pervasive[14], some guideline developers require a verification of the accuracy and completeness of declarations. The verification can be done e.g. by comparing the declarations of the contributors with open-source COI databases or information retrieved by an internet search. If contributors fail to disclose some interests that are later identified from other sources, it may be necessary to take some management measures. The individuals responsible for verification, the verification methods, and measures to deal with inaccurate and incomplete statements should be described (e.g., in the conflict of interest policy of the guideline development organization).

**Example:** *“The American Academy of Neurology (AAN) policy stipulates that information provided on completed Forms may be compared to information publicly available through the Open Payments program to verify the disclosed interest.”[6]*

**Item 13:** Describe the criteria used for assessing whether an interest qualifies as a COI and any assessment of the level of risk associated with the COI.

**Explanation:** Assessing whether an interest qualifies as a COI can help with COI management. One judgement is whether the individual’s declared interests create a significant risk of inappropriately influencing that individual’s duty in the development of the guideline. Some developers assess the level of risk associated with the COI, that is, the level of the influence of the interest on the guideline development process.

**Example:** *“This assessment of disclosed relationships for possible COIs was based on the relative weight of the financial relationship (ie, monetary amount) and the relevance of the relationship (ie, the degree to which an independent observer might reasonably interpret an association as related to the topic or recommendation of consideration).”[15]*

**Item 14*:** Report the results of the assessment of whether the declared interests were considered COI.

**Explanation:** The individuals or committee responsible for COI management determine whether an interest constitutes a COI and, if applicable, the level of the COI, based on the criteria for assessing the interest presented in the previous item (13). The details of this assessment should be reported in the guideline. The reader can compare the assessment with the criteria mentioned in the policy to evaluate the appropriateness of the assessment.

**Example:** *“Managing declarations of interest and conflicts of interest.*

*All contributors declared no interests, with the exception of those listed below. A summary of declared interests and how they were managed is provided:”[16]*

| Name | Declaration of Interest | Conflict of interest and management |
| --- | --- | --- |
| GDG Members | | |
| Capucine de Fouchier | Change of employment | **No conflict of interest identified** |
| [name of the individual] | Participation in evidence reviews for key questions (4,5,6,7,13) | **Significant conflict of interest identified.** A conditional participation management plan was initiated. Professor [name of the individual] participated as a member of evidence review team on discussions about the PICOs, not as a member of the GDG; therefore he had no vocal rights or voting right for recommendations 4,5,6,7 and 13. |

**Item 15:** Describe the COI management strategy and how (if applicable) it accounts for the level of the risk associated with the COI [e.g., requiring a minimum percentage of panelists free from COI, exclusion from the panel, exclusion from specific roles (e.g., chair, systematic reviewer), exclusion from specific aspects for the process (e.g., voting), divestment, restriction from relations that could lead to COI during/after assignment]

**Explanation:** The management strategy of COI is the key to effective avoidance or reduction of the influence of COI. Effective management requires different actions according to different levels of COI, that is, management strategies that reflect the level of risk the COI poses on the guideline. For example, in case of a severe COI, it may be necessary to exclude the individual from the entire development process, whereas in the case of a less severe COI it may be sufficient to restrict the individual’s participation in some specific activities.

**Example:** *“The description and suggested actions for disclosing interests are given in Table 6-2 below.”[10]*

*Table 6-2. Description of disclosures and recommended actions.*

| Level | Type of Disclosure | Range of Possible Recommended Actions | Description |
| --- | --- | --- | --- |
| **1** | \| - No financial disclosures of any value - No nonfinancial disclosures that would impact the judgment of the Task Force member   Financial Interests that do not need to be disclosed:   - Income from seminars, lectures, teaching engagements, or service on advisory committees or review panels for public entities or nonprofit organizations that do not have a vested interest in the specified topics - Diversified mutual or retirement funds   Nonfinancial Interests that do not need to be disclosed:   - Employment from nonprofit organizations such as government agencies and nonprofit entities that do not have a vested interest in the specified topics - General membership in a professional society - Attendance at presentations or conferences related to the topic(s) of interest \| \| --- \| | No Action | No disclosure or recusal necessary |
| 2 | - Providing public comments, expert testimony, or participation in speaking bureaus on a relevant topic (excluding speaking engagements on behalf of a product) - Any relevant financial disclosure valued at $1,000 or less - Participation in any governmental organizations, professional societies, or other organizations (as an officer, medical staff, board member, director, expert advisor, or consultant) related to the topic(s) of interest - Serving as editor or deputy editor of an academic journal, book, or Web site | Information disclosure to Task Force only. | Member may participate as primary lead, and may discuss and vote on the topic |
| 3 | - Any relevant financial disclosures valued at more than $1,000 - Participation in any proprietary companies (as an officer, medical staff, board member, director, expert advisor, or consultant) related to the topic(s) of interest such that the member would stand to gain financially from a specific outcome of a recommendation statement. - Speaking engagements on behalf of a product - If a member has significant nonfinancial interests in a specific outcome of a recommendation statement - The member has one or more publications or research grants that are likely to be part of the evidence review, and that address key questions in the analytic framework, or that express opinions related to the topic. Whether or not action is needed will depend on the specific content of the publications and/or grants, and the source of funding of any grants. | Possible exclusions from Task Force roles as a result of Level 3 disclosures  Include:  Member may not serve as primary lead for topic workgroup | Member may not participate as the primary lead of the topic workgroup specific to the conflict, but may serve as a lead on the topic workgroup and discuss and vote on the topic. |
|  |  | Member may not serve as the primary spokesperson for the topic | Member may not participate as the Primary spokesperson for the topic specific to the conflict, but may serve as a lead on the topic workgroup and discuss and vote on the topic. |
|  |  | Member may not serve as a lead on the topic workgroup | Member may not participate as a lead in the topic workgroup specific to conflict, but may discuss and vote on the topic. |
|  |  | Recusal from all participation in topic activities | Member may not participate as a lead on the topic workgroup specific to conflict and may not discuss or vote on the topic. Member will leave the meeting room for all discussion and voting. Publicly released recommendations will denote the member's recusal from participation and voting on this topic. |
| The member may choose to disclose to the Task Force chairs either a strongly held opinion that results in the potential for bias, or a personal or family illness that may lead to bias but which should be held confidential. This may result in recusal from a particular topic, at the discretion of the Task Force chairs.  If a relationship could be classified in more than one level (for example, service as a medical editor [Level 2] that is compensated at more than $1,000/year [Level 3]), it would be classified at the higher level (Level 3, in this case). | | | |

**Item 16:** Describe any implications for non-compliance with rules of declaration.

**Explanation:** Non-compliance with the declaration of interest rules may negatively impact the trustworthiness of the guidelines. If it becomes known that a contributor failed to comply with the declaration policy, actions may need to be taken (e.g. the exclusion from any activities). These actions should be pre-defined (e.g., in the policy) so that they can later be applied objectively during the actual guideline development process.

**Example：***“An AAN guideline developer’s or reviewer’s failure to accurately, honestly, and fully complete the Relationship Disclosure Form or adhere to the responsibilities described in this Implementing the AAN Conflict of Interest Policy for Guidelines and Case Definitions section of the manual may face sanctions by the AAN, including any or all of the following:*

*• Exclusion from developing future AAN guidelines or case definitions*

*• Exclusion or removal from participation on AAN boards, committees, subcommittees, work groups, task forces, guideline or quality measurement panels, or other AAN positions*

*• Disciplinary action under the AAN’s Disciplinary Action Policy at AAN.com/membership/professionalism-and disciplinary-program/”[6]*

**Item 17:** Describe any process to resolve disputes in the implementation of the COI policy.

**Explanation:** In some cases, there may be disagreements between the members of the committee managing the declarations and conflicts, or the contributors may find that their interests have been unfairly assessed. A COI policy should therefore describe a clear action plan to resolve disputes in a fair and transparent way e.g., through internal communication and negotiation, or by seeking mediation from an independent third party.

**Example:** *“When considering Relationship Disclosure Forms and possible courses of action or in instances of disagreement between a Person and the Reviewing Authority, any Reviewing Authority may consult with the President. The President may (at the President’s discretion) bring such matters to the Executive Committee for review and decision-making. The Executive Committee may consult with the Ethics, Law and Humanities Committee.”[6]*

**Item** **18*:** Report the results of the COI management strategy (e.g., whether individuals were excluded or their contribution was restricted)

**Explanation:** Typically, the. individual or committee responsible for COI management decides (according to the policies addressed in item 15) whether to restrict a person's participation in a guideline based on the results of the COI assessment. Restrictions can include key steps such as exclusion from guideline development or not being allowed to participate in the consensus on certain recommendations. Explicitly reporting the exact actions that were taken to manage each individual COI is key for readers to understand the potential influence of the COI on the recommendations and confirm that the COI policy was followed adequately.

**Example:** *“Below is a summary of the Declared conflicts of interest and how these were managed.*

*Dr [name of the individual] declared in her DoI form that she had received research support routed via her University on various research projects on dementia in 2015 and 2016. These amounts were used to pay her salary by the University. She also declared receiving a payment of US$1000 in 2017 to advise a pharmaceutical company, [name of the company]. The topic was on the availability of data on dementia costs for a medicine that has since been withdrawn due to lack of effectiveness.*

***Action:*** *It was felt that this interest is insignificant or minimal and unlikely to affect, or be reasonably perceived to affect, Dr [name of the individual]’s judgement in the development of the present guidelines. No further action was necessary.”[3]*

**Section 2:** **Funding of the guideline project**

**Item 19*:** Indicate which funding policy was implemented (e.g., the organization's funding policy, policy developed specifically for the guidelines), and how to access it.

**Explanation:** As with the COI policy (see Item 1), if a specific funding policy (either of the developing agency, another adopted published policy, or a funding policy developed specifically for the guideline) is followed, it should be reported in the guideline including a reference (link, bibliographic details).

**Example:** *“The American College of Cardiology (ACC) guideline reported the policy on compliance with funding, which are available at: https://www.acc.org/guidelines/about-guidelines-and-clinical- documents/relationships-with-industry-policy.”[17]*

**Item 20:** Indicate whether funding should not be accepted from specific sources, if applicable.

**Explanation:** Considerations for not accepting funds from a specific source include legality (i.e., funds from illegal or criminal activities), public health harm (i.e., funds from sources associated with the manufacture or promotion of products harmful to health), morality and ethics (i.e., funds from activities contradicting generally recognized moral and ethical values), as well as reputation and image (i.e., funds from sources whose activities are incompatible with the developer organization's or contributors’ values). Similarly, accepting funds from sources with interests related to the topic of the guideline may interfere with the process and influence the final recommendations. For example, guideline funding policies could state that guidelines are not permitted to receive funds from manufacturers of drugs that can be used to treat the guideline’s target disease.

**Examples:** *“WHO guidelines cannot be funded, in whole or in part, by private sector entities or by non-state actors that are not at “arm’s length” of a private sector entity. Private sector entities are commercial enterprises. That is to say, they are businesses that are intended to make a profit for their owners. The term also refers to entities that represent private sector entities, or are governed or controlled by private entities.” [4]*

*“The European Society of Cardiology (ESC) and European Respiratory Society (ERS) guidelines specify that its entire financial support for guideline development comes from the ESC and ERS without any involvement from the health care industry.” [18]*

**Item 21:** Indicate whether the amount of funding should be reported.

**Explanation:** The amount of funding may correlate with the risk of influencing the content of the guideline and it is assumed that reporting the amount of funding increases transparency and public trust. Some funders may require recipient organizations to report the amounts of funding to ensure transparency and effectiveness of the use of the funding, or to meet the funder's own compliance and reporting requirements. It should be noted that reporting the amount of funding needs to comply with relevant laws and regulations, policies and ethical principles.

**Example:** *All sources of funding for the guideline should be reported, including the exact amount of funding received. [Self-made example]*

**Item 22*:** Report whether the guideline received or is expected to receive funding, whether direct or indirect (if not, items 23-25 are not applicable)

**Item 23*:** Provide the name(s) of the direct or indirect funder(s)

**Item** **24*:** Provide the identifiers for the funding (e.g., grant number), if applicable.

**Item** **25*:** Indicate whether the funder(s) set any restrictions on how to use the funding.

**Explanation:**

Detailed information on the funder of the guideline is essential to identify the source of funding and to assess the potential impact of the funding on the guideline. Direct funders provide funding for the guideline itself, while indirect funders provide funding for guideline development organizations who in turn may use it for guideline development projects. The names and details of known funders should be reported in the guideline. Since the number of indirect funders is often high, the guideline development organization could report the details of such funders on the official website or platform, and the guidelines can in turn include a link to this website. The identifier of the funding may be a name, number, or other indicator that identifies the specific funding program or grant.

Funders may place restrictions on the use of funds. If such restrictions are placed, they should be reported in the guidelines. If no restrictions are imposed, it is also recommended that the absence of restrictions is explicitly stated to increase transparency and credibility.

**Example:** *“Funding information: National Natural Science Foundation of China, Grant/Award Number: 82171710; National Key R&D Program of China, Grant/Award Numbers: 2021YFC2701700, 2021YFC2701704.”[19]*

**Item 26*:** Describe the role of funder(s) in the different steps of guideline development, planned dissemination and planned implementation.

**Explanation:** Guideline developers should report on the role of funding in the different steps of guideline development and dissemination to allow users to assess the potential influence of the funder on the process. Potential roles include planning, topic prioritization, question development, review of the evidence, decision to publish, and dissemination expenses.

***Example:*** *“The guideline was developed and funded by European Society of Human Reproduction and Embryology (ESHRE), covering expenses associated with the guideline meetings, with the literature searches and with the dissemination of the guideline.”[20]*

*“Development of these guidelines was wholly funded by the 4 collaborating organizations: ASH, ISTH, NHF, and WFH. Organization staff supported panel appointments and attended meetings but had no role in choosing the guideline questions or determining the recommendations.”[21]*

**Item 27*:** Describe any mitigation strategies (e.g., use of a funding firewall) to minimize the influence of the funder(s) on the guideline development process.

**Explanation:** To minimize the impact of funding on the development process, a variety of strategies can be used, such as: the use of a funding firewall, meaning that the funding sources are not disclosed to (at least some) contributors during the guideline development process; or by prohibiting any representative of the funder to have access to any part of the guideline development process. It would be important to report on any strategies used by the guideline developers.

**Example:** *“The American Thoracic Society (ATS) and the European Respiratory Society (ERS) state that in the absence of public funding, firewalls should be erected to insulate guideline development from the potential for or appearance of industry bias. Ideally, clinical practice guidelines should be developed without commercial support.”[22]*

1. **Conclusion**

This document explains in detail the rationale behind each item in RIGHT-COI&F and gives examples from published guidelines and COI and funding policies on adequate reporting. For one item (21) we found no suitable examples, and these aspects of COI and funding need particular improvement in the future. This document could be used as training and explanation material for the RIGHT-COI&F checklist, aimed at both guideline developers and guideline users. Using this document, stakeholders can deepen their understanding of the items of the RIGHT-COI&F checklist, in order to report COIs and funding in guidelines accurately and completely, ultimately contributing to improving the overall quality and transparency of guidelines. The RIGHT-COI&F working group will continue to evaluate and improve RIGHT-COI&F based on the latest evidence, suggestions and opinions from the feedback, and new scientific developments. The checklist including the explanation and elaboration document will be updated as necessary.

References

1. Mother D, Schulz KF, Simera I, et al. Guidance for developers of health research reporting guidelines[J]. PLoS Medicine, 2010,7(2): e1000217.

2. Chen Y, Yang K, Marušic A, et al. A reporting tool for practice guidelines in health care: The RIGHT statement[J]. Annals of Internal Medicine, 2017,166(2): 128-132.

3. World Health Organization. Risk reduction of cognitive decline and dementia: WHO guidelines[M]. Geneva: World Health Organization, 2019.

4. World Health Organization. WHO handbook for guideline development, 2nd Edition [EB/OL]. (2014-12-18)[2023-08-17]. <https://www.who.int/publications/i/item/9789241548960>.

5. The American Academy of Family Physicians. Clinical Practice Guideline Manual [EB/OL]. (2017-12-01) [2023-08-17]. <https://www.aafp.org/family-physician/patient-care/clinical-recommendations/cpg-manual.html>.

6. American Academy Neurology. Clinical Practice Guideline Process Manual (2017 Edition) [EB/OL]. (2017-06-17) [2023-08-17]. <https://www.aan.com/siteassets/home-page/policy-and-guidelines/guidelines/about-guidelines/17guidelineprocman_pg.pdf>.

7. Development Process for Practice Guidelines of the American Psychiatric Association – Revised [EB/OL]. [2023-08-17]. <https://www.psychiatry.org/File%20Library/Psychiatrists/Practice/Clinical%20Practice%20Guidelines/Guideline-Development-Process.pdf>.

8. Infectious Diseases Society of America. Handbook for Clinical Practice Guidelines Development. [EB/OL]. [2023-08-17]. <https://www.idsociety.org/globalassets/idsa/topics-of-interest/lyme/idsa-handbook-on-cpg-development-10.15.pdf>.

9. Qaseem A, Wilt T J, Clinical Guidelines Committee of the American College of Physicians*. Disclosure of interests and management of conflicts of interest in clinical guidelines and guidance statements: methods from the Clinical Guidelines Committee of the American College of Physicians[J]. Annals of internal medicine, 2019, 171(5): 354-361.

10. U.S. Preventive Services Task Force. Procedure Manual[EB/OL]. [2023-08-17]. <https://uspreventiveservicestaskforce.org/uspstf/about-uspstf/methods-and-processes/procedure-manual/procedure-manual-section-1#7>.

11. Resuscitation Council (UK) Conflict of Interest policy. [EB/OL]. [2023-08-17]. <https://www.resus.org.uk/sites/default/files/2020-06/Conflict%20of%20Interest%20-%20Policy.pdf>.

12. Steinberg, E., Greenfield, S., Wolman, D. M., et al. Clinical practice guidelines we can trust[M]. Washington (DC): National Academies Press (US), 2011.

13. World Health Organization. WHO guidelines for the prevention of sexual transmission of Zika virus[M]. Geneva: World Health Organization, 2020.

14. El-Rayess H, Khamis A M, Haddad S, et al. Assessing concordance of financial conflicts of interest disclosures with payments’ databases: a systematic survey of the health literature[J]. Journal of Clinical Epidemiology, 2020, 127: 19-28.

15. Johnson S, Lavergne V, Skinner A M, et al. Clinical practice guideline by the Infectious Diseases Society of America (IDSA) and Society for Healthcare Epidemiology of America (SHEA): 2021 focused update guidelines on management of Clostridioides difficile infection in adults[J]. Clinical infectious diseases, 2021, 73(5): e1029-e1044.

16. World Health Organization. WHO guidelines on mental health at work[M]. Geneva: World Health Organization, 2022.

17. Heidenreich P A, Bozkurt B, Aguilar D, et al. 2022 AHA/ACC/HFSA guideline for the management of heart failure: executive summary: a report of the American College of Cardiology/American Heart Association Joint Committee on Clinical Practice Guidelines[J]. Journal of the American College of Cardiology, 2022, 79(17): 1757-1780.

18. Humbert M, Kovacs G, Hoeper MM, et al. 2022 ESC/ERS Guidelines for the diagnosis and treatment of pulmonary hypertension: Developed by the task force for the diagnosis and treatment of pulmonary hypertension of the European Society of Cardiology (ESC) and the European Respiratory Society (ERS). Endorsed by the International Society for Heart and Lung Transplantation (ISHLT) and the European Reference Network on rare respiratory diseases (ERN-LUNG)[J]. European heart journal, 2022,43(38): 3618-3731.

19. Li Y, Hu Y, Chen Q, et al. Clinical practice guideline for kangaroo mother care in preterm and low birth weight infants (2022)[J]. Journal of Evidence‐Based Medicine, 2022.

20. ESHRE Guideline Group on RPL, Bender Atik R, Christiansen OB, et al. ESHRE guideline: recurrent pregnancy loss: an update in 2022[J]. Human reproduction open, 2023, 2023(1): hoad002.

21. James P D, Connell N T, Ameer B, et al. ASH ISTH NHF WFH 2021 guidelines on the diagnosis of von Willebrand disease[J]. Blood advances, 2021, 5(1): 280-300.

22. Boyd E A, Akl E A, Baumann M, et al. Guideline funding and conflicts of interest: article 4 in integrating and coordinating efforts in COPD guideline development. An official ATS/ERS workshop report[J]. Proceedings of the American Thoracic Society, 2012, 9(5): 234-242.
