# Supplementary material for: Reporting Conflicts of Interest and Funding in Healthcare Guidelines: The RIGHT-COI&F Checklist
Source: Ann Intern Med. Author manuscript; Available in PMC 2024 Dec 24. (PMC7616250; doi:10.7326/M23-3274)
Supplement: Supplemental file 3 [file EMS196956-supplement-Supplemental_file_3.docx]

**Supplement 3. Summary of feedback given during the consensus meetings with responses from the coordination team**

The comments are categorized according to different themes and sections. Please note that the item numbers may not correspond to the final version of the checklist.

***Length of the checklist***

Comments:

- We should keep the checklist to the minimum.

**Response:** Individual guidelines need to follow only the implementation related items if their organizational policy adheres to the checklist. Therefore, the number of applicable items for most guidelines is feasible.

***Neutrality in giving recommendations about the actual management of conflicts of interest (COI) and funding***

Comments:

- How neutral should we be – this is a reporting checklist, but should we also include hints about appropriate management strategies?

**Response:** This is a reporting checklist where each item provides a recommendation on how to report on a given issue. We will not suggest any specific measures (even indirectly) about how interests should be declared, or how COI and funding should be managed. We will however also avoid giving any examples that are not in line with appropriate guideline development methodologies (for example, oral declaration was removed from the examples in one of the items for this reason).

***Introduction and general principles for the checklist***

Comments:

- The definition of interest: almost anything (for example expertise in a topic) can be an interest – the example of religious beliefs may need rethinking.
- Interests such as beliefs and opinions have a different nature than financial interests (financial interests either exists or not, whereas everyone has some beliefs and opinions that may have an influence).

**Response:** We agree that almost anything can be an “interest” in the sense of the definition we use. We also agree that financial and intellectual interests are of different natures which means that they need to be managed in different ways. The examples we gave do not imply that all such interest should be declared – in other words, in our view religious beliefs, political opinions, etc. are interests, but how such interests are classified and which types of interests need to be declared depends on the organization's policies. We have given only examples without the intention to cover all different types of interest. To avoid confusion, we will revise the example in the final version of the paper.

***Distinction between policy- and implementation-related items***

Comments:

- Should we have two separate checklists (for organizations and authors), or keep both in the same checklist?
- The term “implementation” may be confusing in this context.

**Response:** We prefer to keep only one checklist, but can consider making different editions or layouts to be used in different situations (e.g. development and updating of organizational policies; development of guidelines). We will reconsider the term “implementation” (we will keep the term “implementation” for now, but will consider also alternative terms such as “application” and will bring this into discussion when circulating the manuscript).

There exist several different situations in which different items of the checklist are applicable:

1. When developing **organizational COI and funding policies**, the authors should report according to the policy items of RIGHT-COI&F checklist.
2. When developing a **guideline**:
   1. If the guideline follows an organizational policy that has been developed according to (and/or verified to adhere to) the RIGHT-COI&F checklist, it is sufficient for the authors to report according to the implementation items of RIGHT-COI&F.
   2. If the guideline follows an organizational policy that has not been verified to adhere to RIGHT-COI&F, the authors should report according to the implementation items of RIGHT-COI&F, but we also recommend to check that the policy adheres to the policy related items.
   3. If the guideline developer does not have an organizational policy, the authors should report according to the entire RIGHT-COI&F checklist.

The details on when to use which items of the checklist will be explained in the final paper.

***Definitions***

Comments:

- There were questions/requests to define several terms, for example, guideline (do we also include evidence reviews), committee, indirect and direct funding, guideline development group

**Response:** We will include a glossary in the publication that explains all terms used in the checklist.

***Relation to the original RIGHT statement***

Comments:

- It is unclear whether and when guideline developers should use the RIGHT-COI&F checklist and when the main RIGHT checklist is enough.
- The use of RIGHT-COI&F should be made mandatory and the original RIGHT checklist updated so that the implementation items of RIGHT-COI&F are merged/linked with RIGHT.
- It would be interesting to analyze (after the completion of the checklist) guidelines that adhere to the original RIGHT but not RIGHT-COI&F, to understand the added value and impact of this checklist (which would be helpful for example for journals)

**Response:** RIGHT is the minimum standard: all guidelines should adhere to the main RIGHT statement, including the two items (18 and 19) related to funding and COI. These two items are practical implementation related items.

The RIGHT working group is planning to update the original version. The RIGHT-COI&F checklist will be also referenced in these items of the updated RIGHT checklist, so that the authors can find further clarification when they need more detailed and specific guidance on reporting the implementation of the COI and funding policies. That is, RIGHT-COI&F is intended to be complementary to, and not a replacement of, the COI and funding related items of the RIGHT checklist. Future research is planned to analyze and explore the added value and impact of the RIGHT-COI&F checklist (e.g. guidelines that adhere to the original RIGHT but not RIGHT-COI&F).

We will also add a more specific clarification on when and how to use RIGHT-COI&F checklist in the background of the final paper.

***Comments on individual items***

Comments:

- Item 3 *[State whether a committee independent of the guideline development group is tasked with implementing the organization’s COI policy, and describe the procedure of setting up this committee (e.g., standing or ad hoc) and its composition]* could be revised to avoid the use of “whether” (items hat can be answered simply by “yes” or “no” should be avoided)
- Does Item 10 *[Report the declarations of interests (initial ones and any updates), including declarations of ‘no interest’]* refer to the full declarations or only summaries; and should this be publicly available, or available only e.g. to the journal editors? For example, in the WHO the original declarations are confidential.
- Item 19 *[Indicate which COI policy was implemented (e.g., the organization’s COI policy, policy developed specifically for the guidelines), and how to access it]* could be moved before item 10.
- COI and funding sections could be in the reverse order (to be in line with the main RIGHT checklist) *[Report the declarations of interests (initial ones and any updates), including declarations of ‘no interest’]*

**Response:** We have revised item 3 (see the revised checklist). For item 10, we request that at least summaries of the interests (all interests, not only those resulting in COI) should be made publicly available. The original DOI documents can be either be made publicly available or kept confidential depending on the organization’s policy. We also suggest specifying that all contributors have their declarations publicly available, and request to report how to access this information, even for interests not determined as COI.

We agree that item 19 could be presented earlier in the checklist. However, we want to order the items primarily according to the topic. Item 19 (as well as item 27 *[Indicate which funding policy was implemented (e.g., the organization's funding policy, policy developed specifically for the guidelines), and how to access it]* in the Funding section) belongs to the topic of public access to information, which in our opinion suits best at the beginning of the respective sections. We, therefore, have decided to move these items to the top of each section.
