# Supplementary material for: Reporting Conflicts of Interest and Funding in Healthcare Guidelines: The RIGHT-COI&F Checklist
Source: Ann Intern Med. Author manuscript; Available in PMC 2024 Dec 24. (PMC7616250; doi:10.7326/M23-3274)
Supplement: Supplemental file 4 [file EMS196956-supplement-Supplemental_file_4.docx]

**Supplement 2. Comments and median scores from the Expert panel with responses and suggested revisions**

| **Section/Topic** | **Items and responses** | **Comments** | **Median** |
| --- | --- | --- | --- |
| **Conflicts of interest (COI) of contributors to the guideline project** | | |  |
| Definitions | **1. State the definition of COI used by the organization *(Policy related item)*** | **C1**-Which organization? I suggest to clarify by writing a further clarification like one or more of the following:-".... by the organization where the respondent is a full-time or part-time employer". Or" ....by the guideline developer organization" Or ".... by the organization where the guideline is intended for implementation" Or Maybe all three descriptions together if applicable. Also add an statement: (if there is a relevant policy in the organization) **C2**- RIGHT already says to describe how COI were evaluated and where to find the disclosures. **C3**- Unclear what organization refers to. Guideline producing organization? **C4**- It is important to capture how organisations define a COI, as it may vary between one organisation and another one. **C5**- The definition of COI is important background but generally additional operational guidance is needed beyond a definition, so people know what to declare. **C6**- Understanding of what is considered CoI varies across Organizations and having a common understanding and definition is critically important. **C7**- "I strongly agree that this is essential for guideline producers (e.g. knowing what/when/why to disclose interests and manage COI) and also for guideline users in terms of interpreting disclosures and holding guideline developers accountable. I might suggest going a step further and providing a suggested definition of COI such as the one provided in the preamble to this survey. In my experience developing and evaluating COI policy, definitions of COI vary widely and policy developers are not always aware of the implications of the variability for implementation. Providing a clear, well-defined, and easily operationalized definition (such as the one above) may provide a useful tool to organizations." **C8**- Any relationships that could potentially bias responsibility and/or judgements of medical professionals can be interpreted as COI. | 7 |
|  | Total number of comments received: 8  **Response to comments**:  We agree that the item needs clarification (by organization, we mean the guideline developer organization). Management and operational guidance will be covered by later items. We will provide a COI definition in the paper reporting the checklist extension. Since item 2 will be deleted, we propose to add also the categrorization of COI into this item. We will clarify in the text for item 1 what categorization refers to.  **Revisions suggested:** We propose to change the wording into **"State the definition and categorization of COI used by the guideline developer organization *(Policy related item)*"**. Categorization is added because item 2 is suggested to be deleted. |  |  |
|  | **2. Describe the categorization used for the different types of interests (e.g., financial, non-financial interests) *(Policy related item)*** | **C1**- I wonder whether it is enough to know the categorization. If there is a statement that there are financial and non-financial interests being two categories, I still do not know how they are defined. However, I acknowledge that this might be too detailed. **C2**- This is a little vague and unclear. Would this not be in the definition? **C3**- Perhaps further detail needs to be provided in non-financial interests, as this tends to be confusing. **C4**- This may however be difficult as there is likely some overlap between these. **C5**- This is critical. The definitions help tremendously. I would consider giving examples of non-financial interests **C6**-More importantly, I think the policy should clearly state the primary interest or duty that is at stake. For example, the WHO Handbook on Guidelines clearly outlines something to do with promoting the health of member state populations - it is specific, measurable, and easily operationalized. Without a clear primary interest/duty explicitly named, identifying relevant secondary interests and evaluating the existence of a COI is highly challenging.  I also do not find the financial/non-financial dichotomy very useful as non is not very descriptive, and both financial/non-financial aspects are often involved.  Instead, after specifying a primary interest, the item might ask policy developers to define the scope of relevant interests that is principle-based (rather than creating trying to create a taxonomy). For example, all relationships (of any kind) with a commercial or other entity with a material stake in the outcome of the guideline. **C7**- Could be unclear what categorization means in relation to the item 1. Do all institutional policies have such categorisation? **C8**- Financial / non-financial; Direct / indirect; Individual / institutional; Intelectual / non-intelectual | 7 |
|  | Total number of comments received: 8  **Response to comments:** The intention was not to propose definitions of financial and non-financial conflicts, but only to address the reporting of clear definitions for any categorization of interests. However, as item 1 already addresses the definition of interests it may be clearer to merge the categoritation into that item.  **Revisions suggested:** We propose to **delete this item** and merge its contents into items 1 and 7. |  |  |
| Preparations for COI management （Original name: COI committee or review body） | **3. State whether an independent committee is tasked with implementing the organization’s COI policy, and describe the procedure of setting up this committee (e.g., standing or ad hoc) and its composition *(Policy related item)*** | **C1**-In general this items makes sense. However, I think there is a need to clearify what is meant by INDEPENDENT committee? **C2**-I agree with this idea, but I wonder if it is possible to completely follow up on the COI of each individual even if we set up a committee. **C3**-Where does this come from? It sounds like this is a recommendation that this be done, but I'm not aware of it. **C4**-What about managing the organization's policy. I.e. in some organizations a central unit will collect disclosure forms for all guideline authors and decide whether some should be excluded. Also maybe describe whether organization has any procedures of verifying disclosures. For example, comparing them to information in public disclosure database. **C5**- In the case there is an independent committee, also provide evidence or their methods and processes regarding reaching a decision regarding an individual COI assessment. **C6**-I wonder whether including in addition to the stating the procedure, also stating who assigns the individuals to the committee **C7-**This is essential for organisations whose task is to develop guidelines, but may be challenging for other institutions/ organisations whose primary role is not guideline development. | 6 |
|  | Total number of comments received: 7 **Response to comments**: By independent we mean a committee that is specifically set to manage COI and not part of the guideline development group. Such a committee is commonly mentioned in guideline handbooks. The reporting of methods and processes of verification and management of COI is included in the other items. As this is a reporting guideline, we also do not intend this as a recommendation, but just a requirement to report whether such a committee existed or not (thus the formatting "State whether..."). The process to assign individuals to the committee is part of the setting up process and thus covered by this item. **Revisions suggested**: We propose to change the wording into**" State whether a committee independent of the guideline development group is tasked with implementing the organization’s COI policy, and describe the procedure of setting up this committee (e.g., standing or ad hoc) and its composition *(Policy related item)*"** |  |  |
|  | **New item (generated from "suggested the new items" below)**: Describe the actions that were taken beforehand to minimize potential COI in the formation of the guideline development group (e.g. screening publicly available DOI/COI databases and inviting only contributors with no potential COI). (Policy related item)   **(Note: This will be discussed in the meeting. )** | **C6 (Move here from the "suggested the new items" section below)** -There is no item about preventing COI or how declarations of interests and evaluation of COI is factored into the formation of a guideline development group. This is crucial for an independent panel and also chair. I would strongly suggest including items that pertain to how COI factored into the selection of guideline committee members and chair and whether the chair was free of COI. |  |
| Declaration of interests | **4. Describe which groups contributing to the guideline project were required to declare their interests (e.g., panelists, systematic reviewers, peer reviewers, staff) *(Policy related item)*** | **C1**- I had assumed everyone needs to adhere, but I suppose it is good to make this clear. **C2**- Alternatively, COI should be applied to all groups. So one could just have a box stating which groups are exempted **C3**- Items 4 and 5 might be made more comprehensive in terms of describing to whom the policy applies - it would make more sense that these target groups both were required to declare their interests AND that COI prevention and management strategies also applied. **C4**- All members involved with the guideline projects | 7 |
|  | Total number of comments received: 4  **Response to comments**: The guideline can report either that all memebers are required to report their interests (and mention exemptions if applicable), or list the groups and individuals who are required to report interests. E.g., WHO GDG (guideline development group) meeting observers do not need to complete a declaration of interests form. They represent a governmental or nongovernmental organization (rather than present in an individual capacity). We have made the statement more comprehensive by referrring to the appliaction of the policy as opposed to only the COI declaration.  **Revisions suggested**: We propose to change the wording into**" Describe to which groups contributing to the guideline project the policy applies (e.g., panelists, systematic reviewers, peer reviewers, staff) (Policy related item)"** |  |  |
|  | **5. Describe whether the individuals declaring their interests were also required to declare the interests of other individuals related to them (e.g., family members) *(Policy related item)*** | **C1**-There is not much wrong with this item. However, cases where this is relevant are unlikely to happen in practice. Considering that too many items might be problematic, I consider this item to be ommitable. **C2**-See comments below. This may be in the standard form or language. **C3**- I worry that these details make it harder for those interested to review the CoI to find the most important information **C4**- Perhaps would be useful to declare or set a certain level of kinship (e.g. spouse, siblings ...) **C5-**The issue with this is the the question is generally restricted to spouses, but not "any family member(s)". Second ,many individuals can. legitimately say that they don't know about the financial/other interests of their family members. | 6 |
|  | Total number of comments received: 5 **Response to comments**: We acknowledge that reporting a large amount of details is time-consuming, but we feel that to ensure the quality of the guideline, such details need to be disclosed. As with other items, this item is not requesting that certain family members should report their interests; it only requires the reporting of the criteria who is required to declare their interests. This is not referring to the names of the individuals. This is a common part of COI declaration forms.  **Revisions suggested:** We propose to change the wording into **"Describe whether the individuals declaring their interests were also required to declare the interests of other individuals related to them and specify who those individuals are (e.g., spouse) *(Policy related item)*"** |  |  |
|  | **6. Describe how the interests were declared (orally or in writing, and whether a standardized form was used) *(Policy related item)*** | **C1**- I am neutral about this. As long as the interests were declared, it should be fine. How it is reported is not a matter to the guideline uses. **C2**- Could ask to provide standard form or language. This would likely cover many of the other issues raised here. **C3**- I worry that these details make it harder for those interested to review the CoI to find the most important information **C4**- If a standardised form was used also provide a copy of it. **C5**- It cannot be described. **C6**- I don't know if this information is needed if there is no actual standard (ie if written declarations are considered neccessary, then naturally, this should be reported). otherwise it is not useful to include this. **C7**-Again, if it's not in writing, nothing counts. This could be a prerequisite **C8**- Writing is the most realistic method. | 6 |
|  | Total number of comments received: 8  **Response to comments**: We feel that although this may seem a minor detail, it is still important to declare the procedure that was used to collect the interests. As this is a reporting checklist, we need to take a neutral approach and avoid giving any recommendations about the actual management process. We however agree that the declarations should be made in writing and have removed the example of oral declaration. It is also out of the scope of the reporting checklist to provide a standard form or language.  **Revisions suggested**:We propose to change the wording into **"Describe how the interests should be declared (e.g., whether a standardized form was used) *(Policy related item)"*** |  |  |
|  | **7. Describe what interests were required to be declared (e.g., according to type) *(Policy related item)*** | **C1**-This item should linked to question 2 with a wizard function. So if item 2 is not answered then item 7 would not be applicable. **C2**-This is a little vague. Perhaps just provide or point to standard disclosure form or language. **C3**-Again, further clarity may need to be provided on non-financial conflicts e.g. (publications, academic career interests) **C4**- Isnt this the same as Q2? **C5**-This item should come up earlier in this sequence **C6**- It is unclear how this differs from #2 - perhaps the definition item can reflect the principle-based approach to defining the primary interest and the relevant secondary interests and this item can list the types of interests that participants must disclose. **C7**-Are the type of interests described? Will this be clearly understood? **C8**-Regardless of classification method, any potential COIs should be disclosed. | 7 |
|  | Total number of comments received: 8  **Response to comments**: The item was intended to cover not only the type mentioned in the previous item 2 (which is suggested to be deleted), but also other aspects. Item 2 has been merged with item 1 to avoid duplication. We will provide examples of types of interests in the explanation and elaboration document**.**  **Revisions suggested**: We propose to change the wording into **"Describe what interests were required to be declared (e.g., according to the type of interest, relevance to the topic, the source of the interest, a minimum amount for financial interest, or the recency) *(Policy related item)"*** |  |  |
|  | **8. Describe what details of the interests (e.g., source, amount, date) were required to be declared *(Policy related item)*** | **C1**- Or just ask to provide standard form or language **C2**- Unclear what is meant by source **C3**- I worry that these details make it harder for those interested to review the CoI to find the most important information **C4**- Describing the source and date of interest is important, but the amount can be categorised by range. However, I am not sure how to determine the points of the amount. **C5**- As above, this information is important only insofar as there are clear thresholds above/below which something is considered an interest/not a conflict. eg any interest within the last 5 years is a common threshold. **C6**- I am not sure including all the details in a long document would make the process more meaningful and honest. **C7**- Any amount of COIs in past three years should be disclosed. | 6 |
|  | Total number of comments received: 7  **Response to comments**: By source we mean the entity with which the interest in question exists. We only ask to provide information on what was requested to be declared, not the declarations themselves (for example, we are not requesting the amounts to be declared, but if the exact amount, range of amount, etc, was required to be declared by the policy, this requirement should be mentioned).   **Revisions suggested**: / |  |  |
|  | **9. Describe any process used for updating declarations of interests (e.g., frequency, schedule, format, procedure to remind/collect the updated interests) *(Policy related item)*** | **C1**- Very important time as this issue is often neglected. However, I wonder how a change in an interest or COI (or say any statement made) that occurs will have to be reported. **C2**-Relevant to living guidelines **C3**-This item is extremelly important especially in the case of rapid and living guidelines as they become more of the norm. A specific method for updating DOI, and its collection need to be in place and transparently reported (when was the last assesment performed, when the DOI where collected and any failure in updating) **C4**-The time period during which a conflict is considered is very important as these can change very quickly and within the lifetime of a guideline development process which can take up to 2 years. **C5**- Shouldn't all interest be declared cumulatively for the whole guideline creation process? Not fully clear why such declaration is necessary. **C6**-When judging an eligibility of each member and every year until the publication of guideline. | 6 |
|  | Total number of comments received: 6  **Response to comments**: It is common that declarations are collected only at the beginning of the process: we agree that this is even more relevant for living guidelines, but even for regular guidelines the development process may take years. Therefore, we think it is important to mention whether and how the declarations were updated. E.g., the American Academy of Family Physicians requires each panel member to update any COI (verbally or in person) at each meeting of the guideline development group. The implementation part is now covered by item 10 which mentions updates. **Revisions suggested**: / |  |  |
|  | **10. Report the declarations of interests, including declarations of ‘no interest’ (This includes the declarations from all contributors who are, according to Item 4, required to declare their interests) *(Implementation related item)*** | **C1**-I wonder how do we want this to be reported (at individual level?). Would it be acceptable if it said that there were no interests at all. **C2**- RIGHT says to report how guideline users can access the declarations. Is this different? **C3**-I don't believe it is helpful to report that no interest was declared. **C4**-I don't understand what is asked here. Do you mean where it is reported? **C5**- For both 10 and 11, it may be also important to include the degree to which these declarations are published or are made public and the extent to which they were edited, redacted, or summarized. | 7 |
|  | Total number of comments received: 5  **Response to comments**:By reporting the declarations of interest, we mean providing details of all interests delcared by the guideline development group members (according to the principles specified under Items 7 and 8; and including explictly mentioning which contributors declared having no interests). In our view this information is essential and should be reported clearly in the guideline. Access to the original declaration forms (which may be confidential) is not covered by this item (this is addressed in Item 20 instead).  **Revisions suggested**: We propose to change the wording into **"Report the declarations of interests (initial ones and any updates), including declarations of ‘no interest’. (This includes the declarations from all contributors to whom the policy applies, according to item 4.) *(Implementation related item)*"** |  |  |
|  | **11. Report any update of the declaration of interests (e.g., provide access to both original and updated version) (if applicable) *(Implementation related item)*** | **C1**-Not sure what is meant here. Is this only about an update (item 9) or about a change as a result of the update? **C2**-Would just incorporate into item above. **C3**-The focus should be on the latest reporting **C4**- Again, especially in the case of rapid and living guidelines, this needs to be set up as part of the guideline process. **C5**-The date of the statement may be important when it is updated. **C6**- I don't understand what is asked here. Do you mean where it is reported? **C7**- Needs to be clarified why this is needed. Does it mean that the item 10 has to have time frame for each declared interest? **C8**- Since after the publication of the guideline, it may not be realistic to update it. **C9-**Updating is often missed, but is. vital. | 6 |
|  | Total number of comments received: 9 **Response to comments**: This item refers to updates of interests. It may be clearer to omit this item as the content is already covered to a large extent by item 10.  **Revisions suggested**: We propose to merge this item with item 10. |  |  |
| Assessment of interests | **12. Describe the process used to verify declarations (if any) *(Policy related item)*** | **C1**-The item is relevant, while there might be misunderstandings what the word verify means **C2**-Maybe an example of what verification could be? **C3**-Another critical item. Is there a minimum check of the stated interests? Are they taken at face value? or do the COI committee perform a look up (Google Search...) of the person in question? Declarations at face-value can incur in a risk of underreporting **C4**-This item may be combined with item 13. **C5**-Should give example, such as in the USA the CMS sunshine database **C6**-This item might also suggest that policymakers specify who is responsible for verifying declarations, how this was done, and how discrepancies between sources were dealt with. **C7**-Not clear how realistic this is. **C8**-A use of open-access database such as Open-payment Database should be considered. **C9**-Verification strikes me as the "next level". **C10-**Without this, there is little value in collecting the DoI. | 6 |
|  | Total number of comments received: 10 **Response to comments**: One possibility to verify the interests is to compare the declarations with publicly available disclosure databases or other existing information. The item requests to report the entire process (including e.g. who was responsible, the method of verification, and how discrepancies between sources were dealt with).  **Revisions suggested**: We propose to change the wording into **"Describe any process used to verify the accuracy and completeness of declarations (e.g., who was responsible, the method of verification, how discrepancies between sources were dealt with). *(Policy related item)*"** |  |  |
|  | **13. Describe the criteria used for assessing whether an interest qualifies as a COI and the level of risk associated with the COI *(Policy related item)*** | **C1**-RIGHT already says to describe how COI were evaluated and managed. **C2**-Also should be clear who makes the call. Does author decide or do they disclose all interest and committee decides which should be viewed as COI? **C3**-This needs to be thoroughly described, and unfortunately still missing in many guidelines. **C4**- It is also considered a definition of the level of risk. **C5**-Give example to better understand what is asked here. For exmple, $ thresholds? **C6**-Depending on how COI is defined and policies operationalized, the organization may not consider level of risk. Instead, this might be rephrased as how the magnitude and/or severity (per the IOM) of the COI was assessed or evaluated (which could include a standardized risk assessment). **C7**- The same above. This may turn into a check box exercise. **C8**-There are no accepted criteria, and all COIs should be disclosed, but the COIs of director and vice-directors should be dealt with more rigorously than other members. | 6 |
|  | Total number of comments received: 8 **Response to comments**: This item only addresses the criteria on what interests qualify as COI, not the evaluation process or management. What should be disclosed (C2) is covered by item 7. We prefer to keep the term 'risk' (instead of mangnitude or severity) to keep in line with the adopted definition of COI which refers to 'risk'.  **Revisions suggested**: We propose to change the wording into**" Describe the criteria used for assessing whether an interest qualifies as a COI and for any assessment of the level of risk associated with the COI *(Policy related item)***“ |  |  |
|  | **14. Report the results of the assessment of which declared interests were considered COI *(Implementation related item)*** | **C1**-Does this mean in general or specifically for individuals? Not sure I understand this. I think you mean for individuals, in which case I would combine with description of how the COI was managed. **C2**-Unclear what is meant here...maybe an example? **C3**-Not sure I understand. In my mind there are 2 thresholds: 1) no COI (ie below threshold $), 2) above threshold, but manageable (e.g., recusing to vote) and 3) unmanagable COI **C4**-The question raised for me with this item is the difference between items 10 and 11 and 14. The guidance may want to clarify what constitutes the published/public 'declaration' (i.e. the interests assessed as COI) versus the 'raw data' (i.e. the source declarations). **C5**-If any assessment occurs, that should be reported. | 6 |
|  | Total number of comments received: 5 **Response to comments**:This item is about implementation, not policy; it requests to list which of the declared interests were determined to constitute a COI. **Revisions suggested**: / |  |  |
| Management of COI | **15. Describe the COI management strategy and how (if any) it accounts for the level of the risk associated with the COI [e.g., exclusion from the panel, exclusion from specific aspects for the process, requiring a minimum percentage of panelists free from COI, divestment, restriction from relations that could lead to COI during/after assignment] *(Policy related item)*** | **C1**-Could simplify by listing management options. Thought not sure this is necessary. **C2**-This could also just be fitted in to the organizations COI policy. Not necessary in every guideline. **C3**-Another extremely important element. Another potential aspect is to define if key stakeholders of a given guideline need to be free from conflicts (e.g. panel chairs, guideline development group, systematic reviewers...) **C4**- If an existing standard is used, this should be noted; for example in Australia NHMRC has developed a set of guidelines for management of COI in guideline development. **C5**-The components of 15 are all very important, but I think 13 covers the risk assessment and shoudl account for the fact that organizations may conceptualize 'risk' differently - the severity, magnitude, impact of a COI will differ according to context.  Item 15 may want to first ask policymakers to describe the array of management strategies available and the ways that they fairly and proportionately address COI (per the IOM policy criteria). Then, a separate item should ask guideline developers to report on how this decision tree was applied (implementation item). I would also note that there has been no mention of the prevention of COI in these items, which I think is crucial to developing rigorous, independent guidelines. I suggest an item that asks how interests/COI were assessed during guideline panel formation and selection is very much needed (e.g. efforts to select independent members and chairs). **C6**-1) Exclusion from the panel or judgement process; 2) More than half of the panel should be free from COI | 6 |
|  | Total number of comments received: 6  **Response to comments**: Whereas item 13 addresses whether an interest qualifies as COI, this item covers the details on how COI should be managed If they exist. As before, we do not intend to propose any concrete methods for management, only address the reporting of the methods that were used. The general principles will include the use of existing standards or other policies.  **Revisions suggested**: We propose to change the wording into**" Describe the COI management strategy (if any) and how it accounts for the level of the risk associated with the COI [e.g., exclusion from the panel, exclusion from specific roles (e.g., chair, systematic reviewer), exclusion from specific aspects for the process (e.g., voting), requiring a minimum percentage of panelists free from COI, divestment, restriction from relations that could lead to COI during/after assignment] *(Policy related item)*”** |  |  |
|  | **16. Describe any implications for non-compliance with rules of declaration *(Policy related item)*** | **C1**- Not sure what this about at all. Is this about someone wrongly declaring his interests? Some people might forget details, while other might intentionally omit details **C2**-This could also just be fitted in to the organizations COI policy. Not necessary in every guideline. **C3**-In many cases, missing. I am still not aware of any penalties applied to people reporting or underreporting conflicts (besides exclusion) **C4**-This sounds like it is a subset of enforcement procedures (who is responsible; how enforcement occurs; implications for non-compliance) **C5**-Preferable, but its process should be considered in the future. **C6**-would assume all involved would be compliant | 5 |
|  | Total number of comments received: 6 **Response to comments**: This item covers objectively the consequences for non-compliance: depending on the COI policy these consequences may depend on the severity, intentionality, etc, which should be reported; but as a reporting guideline we do not provide any recommendation on the actual management procedure. Specifying who is responsible and how enforcement occurs are covered by item 3.  **Revisions suggested**: / |  |  |
|  | **17. Describe any process to resolve disputes in the implementation of the COI policy *(Policy related item)*** | **C1**-not fully sure what is meant here **C2**-This could also just be fitted in to the organizations COI policy. Not necessary in every guideline. **C3**-A little less critical, perhaps having some mechanism of appeal or revision of conflicts would be appropriate, again in the context of rapid evolving guidelines. For example if a financial conflict exists, but the participant decide to resolve the conflict (e.g. sell stock of a given company) may prompt to a revision of the conflict and allowing to participate again.) **C4**-I think this item could be merged with the one on the independent oversight committee - this seems part of their mandate. | 5 |
|  | Total number of comments received: 4  **Response to comments**: This item refers to any process when disputes arise (whether between the individual member and the committee, or between committee members). The issue of revision of COI (C3) is covered by item 15 (divestment).  **Revisions suggested**: / |  |  |
|  | **18. Report the implementation of the COI management strategy (e.g., whether individuals were excluded or their contribution was restricted, or any other relevant actions) *(Implementation related item)*** | **C1**- Would combine with assessment item 14 above. **C2**-Seems very similar to #15 **C3**-Very important and again often underreported. This perhaps could be done at a recommendation level, if only specific members were excluded for a given recommendation, or across the guideline. **C4**-The components of 15 are all very important, but I think 13 covers the risk assessment and shoudl account for the fact that organizations may conceptualize 'risk' differently - the severity, magnitude, impact of a COI will differ according to context.  Item 15 may want to first ask policymakers to describe the array of management strategies available and the ways that they fairly and proportionately address COI (per the IOM policy criteria). Then, a separate item should ask guideline developers to report on how this decision tree was applied (implementation item). I would also note that there has been no mention of the prevention of COI in these items, which I think is crucial to developing rigorous, independent guidelines. I suggest an item that asks how interests/COI were assessed during guideline panel formation and selection is very much needed (e.g. efforts to select independent members and chairs). **C5**- It is fair to report it. Also, other guidelines can implement and imitate the management measures. | 6 |
|  | Total number of comments received: 5 **Response to comments**: This item differs essentially from item 14 (which is about declaring whether an interest constitutes COI, not about how the COI were managed thereafter); and from item 15 (which is policy-related, not implementation-related). The item requests a detailed description of how the COI were managed. We agree that in some cases exclusion only from activities related to certain recommendations is appropriate. However the checklist should be applicable to the entire guideline, not only the recommendations, so we suggest to keep the item in its present form.  **Revisions suggested**: / |  |  |
| Public access to the information | **19. Describe whether the organizations’ COI policy is publicly available, and if yes, how to access it *(Policy related item)*** | **C1**- Is not this only relevant if the organization has a firm COI policy. The opposite could be a COI policy only developed for this project. In the latter case, it might be rather unlikely that it is publicly available. **C2**-If this is available, then can they simply refer to this instead where necessary? **C3**-This should be mandatory. Often finding the COI policy is one of the hardest parts and is deeply buried into websites. | 7 |
|  | Total number of comments received: 3 **Response to comments**: We would like to clarify that this should not be restrited to cases where the guideline follows an existing organizational policy, but also for situations where a specific COI policy was developed for the guideline. We also suggest to change this into an implementation related item.  **Revisions suggested**: We propose to change the wording into**" Indicate which COI policy was implemented (e.g., the organization's COI policy, policy developed specifically for the guidelines), and how to access it *(Implementation related item)*“** |  |  |
|  | **20. Report whether the COI declarations of the guideline contributors are publicly available, and if yes, how to access them. If the declarations of interest are confidential, this should be stated *(Implementation related item)*** | **C1**-Isn't this already in the RIGHT statement? **C2**-Another important point, as usually panellists may not be inclined to have their conflicts or interests (especially if financial to be exposed). If they are kept confidential, an important reason should be given, and mechanisms to deal with them clearly stated. **C3**- I am not sure someone with confidential interests should be included. I would love to hear other perspectives. In my mind this counters what we are trying to do here. **C4**-In Japan, we made the open access database to evaluate them. https://yenfordocs.jp/en But, it takes time in preparing the necessary data. | 6 |
|  | Total number of comments received: 4  **Response to comments**: This item refers only to the availability of the original declaration documents (which often are confidential), not to the conflicts themselves (which usually should be publicly disclosed). However, we have removed this item as the content is covered by item 10 **Revisions suggested**: We suggest to **delete this item.** |  |  |
| **Funding of the guideline project** | | |  |
|  | **New item (generated from 21e's comment 2): Indicate whether funding should not be accepted from specific sources, if applicable *(Policy related item)*** |  |  |
|  | **New item (to replace item 21d): Indicate whether the amount of funding should be reproted *(Policy related item)*** |  |  |
| The source of funding | **21. Report the details of the sources of funding of the guideline (both received and expected funding), including a ‘no funding’ status, and report, if applicable: *(Implementation related item)*** |  | 6 |
|  | Total number of comments received: 0 |  |  |
|  | **21a. Names of the funder(s) (e.g., the National Natural Science Foundation)** | **C1**-isn't this already part of RIGHT statement? | 7 |
|  | Total number of comments received: 1 **Response to comments**: RIGHT-COI&F is intended to be usable also as a standalone document, which means there may be an overlap with the main RIGHT checklist.  **Revisions suggested**: As we have merged this with sub-item 21b, we have revised this sub-item as **"Names of the direct or indirect funder(s) (e.g., the National Natural Science Foundation), including if possible details of indirect funders."** |  |  |
|  | **21b. Type(s) of funder(s) (e.g., governmental, for the profit entity, not for profit entity)** | **C1**-not sure this is neccessary. There is also the problem that the type is hardly definable. **C2**-categorization of profit status is not always straightforward. **C3**-Suggest making specific categories, this will make it easier to use **C4**-One issue is that sometimes an agency or professional society is the direct funder, and they might be a non-profit, but they receive funding from a commercial entity. How is this type of situation to be addressed? **C5**-This could also request more detail in terms of type of funder and the entity's sources of funding (e.g. not-for-profit foundations solely funded by commercial entities) | 7 |
|  | Total number of comments received: 5  **Response to comments**: We agree that reporting the type of the entity would require first a clear categorization. As the name of the funder is already declared, this sub-item may not be necessary.  **Revisions suggested**: We suggest to remove this item as the previous item 21a already identifies the funder . |  |  |
|  | **21c. Identifiers for the funding (e.g., grant number)** | **C1**-not sure what an identifier could be if there is no grant number **C2**-(if feasible) As sometimes at this stage of the guideline project there could be a preliminary approval of the funding body but the number is not yet issued or sometimes in other cases the funding body will issue the fund after the project is completed **C3**-if applicable **C4**-In reporting grant number, the rules of the funding agencies should be followed. | 6 |
|  | Total number of comments received: 4  **Response to comments**: We agree that this information may not always be applicable or available (not all forms of funding have an identifier).  **Revisions suggested**: We propose to change the wording into **"Identifiers for the funding (e.g., grant number), if applicable”** |  |  |
|  | **21d. Amount of funding** | **C1**- it is not standard to report it in other projects. Why should this be the case here? **C2**-Could be provided in also in ranges (e.g. >10,000$ >100,000 $) **C3**-Total funding amount might be difficult to report if the organisation has provided support in addition to the funding directed specifically to guideline development. | 6 |
|  | Total number of comments received: 3  **Response to comments**: We agree that the reporting of exact amounts is not standard practice in most reseach, and the amount used to support the guideline may be difficult to determine.   **Revisions suggested**: We propose to replace this sub-item with a policy related item (placed before item 21): **"Indicate whether the amount of funding should be reported (Policy related item)"** |  |  |
|  | **21e. Whether the funding was unrestricted** | **C1**-RIGHT already asks for the role of the funder. **C2**-Statements that 'unrestricted funding' was provided are often meaningless, as this does not prevent sponsor influence. It is much more important to specify what firewalls are in place, if any, to prevent sponsor involvement in guideline development (or more generally the role of the sponsor in guideline development). **C3**-Meta-research studies suggest that authors of research articles often state the funder was not involved even in cases where the funder was very much involved. . . this item might instead ask for a detailed explanation of how the funder was involved and efforts made to ensure independence. **C4**- I think it would be better to state specifically what unrestricted means. | 6 |
|  | Total number of comments received: 4  **Response to comments**: As this is a reporting checklist, we are aiming to take a neutral approach and avoid giving any recommendations on the actual management. Therefore we propose to modify this item so that it requests more details about the restrictions.  **Revisions suggested**:We suggest to revise the item as **"Whether the funder set any restrictions on how to use the funding".** |  |  |
| Management of funding | **22. Describe who managed the guideline funding (e.g., a special committee or group) *(Policy related item)*** | **C1**-I think this item will need further clarification. **C2**-Those managing the funding must also declare their interest and must follow the prior process of identifying whether there is any conflict. **C3**-I was unsure what managed means in this context. Perhaps this could be clarified? | 5 |
|  | Total number of comments received: 3  **Response to comments**: We agree that the item was formatted somewhat unclearly, and it also received a low median score. This item refers only to the management of funding, not as important as having a committe for the management of individual COI. Usually every organization has their internal administrative processes to manage funds, and we think the description of who is doing that no or little added value to the reader. We therefore propose to delete this item.  **Revisions suggested**: We suggest to **delete this item.** |  |  |
|  | **23. Report the different steps of the development of the guideline, including future steps (e.g., dissemination), for which the funding was or will be used *(Implementation related item)*** | **C1**-Already in RIGHT statement **C2**-This seems like an unnecessary extra bureaucratic step to me. The key issue is whether the funding was commercial or not, not which parts of guideline development it was used for. **C3**-This could be useful but likely represents a significant administrative burden and investigation to determine if there was a meaningful CoI that may outweigh the benefits. **C4**-Could this be more open-ended and have a detailed report on what the funding was used for? E.g. if funding staff, a description of the staff's role is more useful than the steps of the guideline development in understanding the scope of the funding. **C5**-Describe how it was used and how it will be used in each step of the guideline development. | 5 |
|  | Total number of comments received: 5  **Response to comments**: We agree that defining how the funding was distributed across the different steps may not always be straight-forward. The information is also essentially covered by the next item that requests to report the roles of funders in different steps. Therefore, we propose to delete this item.  **Revisions suggested**: We suggest to **delete this item.** |  |  |
|  | **24. Report the role of funder in the different steps of guideline development and planned dissemination *(Implementation related item)*** | **C1**-This is very important and whether a statement that reads that the funding body will not interfere with the formulation and articulation of the guideline recommendations. **C2**- Already in RIGHT statement **C3**-Suggest describing the different steps **C4**-Role of funder needs to be very clearly stated, especially if it affects in any way the final content of the guideline or recommendations. **C5**- In my experience, funders of guidelines should not be involved at all and this is an all-encompassing prohibition. It may be possible to manage any interests of a funder, but the benefits/costs of this are outweighed by the transaction costs and risk of perception of bias/conflict. **C6**-There should be no role **C7**-Also if the funder had no role.. | 6 |
|  | Total number of comments received: 7  **Response to comments**: In this item, we mean the authors should describe each step the funder had a role in and describe that role, including the exact ways the funding was used in different steps (e.g. paying salaries, organizing meetings or travel, etc.), whereas item 26 means the influence of the funders on the guideline's content. Also clarify that if the funder had no role, that should be repoted. Therefore, in this case a "positive" response (i.e. declaration of the way how the funding was used) is also appropriate.  **Revisions suggested**：/ |  |  |
|  | **25. Report whether the guideline contributors were aware of the source and other details of the funding (i.e., whether a funding firewall was set or not) *(Implementation related item)*** | **C1**-hard to testify if someone else is aware. **C2**-This statement is unclear and can be read to refer to two different issues: 1) awareness of guideline contributors of funding source; and 2) whether a firewall has been set up. I have stated I disagree with the statement because I think it needs to be more clearly stated. Guideline contributors should of course be aware of the funding source. And a funding firewall is a good idea. **C3**- I had not ever considered this but its an interesting point. Would the purpose be to identify a conflict that is unknown at the time of funding? **C4**-This point is not very clear to me. | 5 |
|  | Total number of comments received: 4 **Response to comments**: We agree that the word "aware" may be misleading; what was meant by this item was a funding firewall i.e. not disclosing the funding sources to the contributors during the process. Contributors would be automatically aware of the (direct) funding of the guidelines.The firewall would however only apply to indirect funding (since the direct funding in most cases would come from the developer organization). Strategies to mitigate the influence of (indirect) funding is in turn covered already by the revised version of item 26, which also mentions firewall as an example. We thus suggest to delete item 25.  **Revisions suggested**: We suggest to **delete this item.** |  |  |
|  | **26. Include a statement that the funding didn’t influence the content of the guidelines, if applicable *(Implementation related item)*** | **C1**-If termed in this way, everybody will include this statement even if not true.  In Germany we have cases where a guideline is funded by a medical society. This society decides on the number of delegates and will always send the highest number of delegates to the guideline committeee. **C2**-Yes this is very important. **C3**-How do we determine whether the funding influenced the content? Are there any criteria? **C4**-This would be implied by the role of the funder. Not sure it's necessary **C5**-meaningless **C6**-Should be clear from step 24 **C7-**Needs to be very clearly reported, if a goverment, funder or agency has any means to influence the content of the guideline and if there are any mechanisms to avoid interference and editorial independence. **C8**-This needs to be related to specific procedures to ensure lack of influence, e.g. firewalls to prevent sponsors from influencing content, as well as policies to prevent authors with funding from the sponsor to be involved. This statement on its own is meaningless. **C9**-This seems meaningless and tokenistic unless accompanied by clear and written rules of accountability should a funder be found to have had a role/influenced the guidelines. But my expectation is that if that is the case, there would anyway be measures taken to rectify the influence of the funder (that would not be contingent of showing that the developers had violated this statement). **C10**-This aligns with my comment above on obtaining interest data for those managing the funds.(comment above:Those managing the funding must also declare their interest and must follow the prior process of identifying whether there is any conflict.) **C11**-This statement in a guideline is completely worthless. If industry sponsors the guideline it does not matter that someone states it didn't influence it. Of course it did. **C12**-Meta-research analyzing funding declaration statements and interviewing clinical trialists suggest that these kinds of statements do not always closely reflect the reality. Instead, a detailed reflexive statement about how independence was maintained may be more meaningful. **C13**-I wonder if making it standard to write had no influence is not necessarily a good disclosure of conflicts of interest. **C14**-Can this be incorporated in 24? **C15**-Yes, but we should consider how no potential influence of funders can be guaranteed. | 6 |
|  | Total number of comments received: 15  **Response to comments**: We fully agree with the comments: as it should be standard practice that funders do not influence the content of the guideline and recommendations, it is likely that the guideline developers will make such a declaration even if it was not true in reality. We therefore suggest to reformulate the item so that it requests to list some concrete actions that were made by the guideline development group to ensure that the funders did not influence the content.  **Revisions suggested**: We propose to revise this item as **"Describe any mitigation strategies (e.g., use of firewall) to minimize the influence of the funder(s) (whether providing direct or indirect funding) on the guideline development process *(Implementation related item)* "** |  |  |
| Public access to the information | **27. Report whether the organizations’ funding policy and documents or additional details on funding are publicly available, and if yes, how to access them *(Implementation related item)*  Revision suggested:** We propose to change the wording into" **Indicate which funding policy was implemented (e.g., the organization's funding policy, policy developed specifically for the guidelines), and how to access it (I*mplementation related item)"*** |  | 6 |
|  | Total number of comments received: 0 |  |  |
|  |  |  |  |
| **Suggested new items** | | |  |
|  | Total number of suggested new items received: 8 **We suggest to add the following the item:** 1. **Describe the actions that were taken beforehand to minimize potential COI in the formation of the guideline development group (e.g. screening publicly available DOI/COI databases and inviting only contributors with no potential COI).** ***(Policy related item)* (Note: This will be discussed at the meeting. )**   **Why some suggested new items were not adopted:** We decided not to include new items that were out of, or too specific, for the scope of RIGHT-COI&F (RIGHT-COI&F is a reporting checklist, not guidance for COI management); unclear; already covered to a large extent by existing items of RIGHT-COI&F; or not feasible in practice to report. For example, reporting relationships with industry (see suggested item 3. under C8) is already covered by items 7-8 (policy) and 10 (implementation). | **C1**-Would it make sense to group by policy vs implementation items instead? **C2**-It is becoming more common that for profit funding supports educational activities or provide funding for entities which can not be tracked on open payment systems. some guidance about what should be included under financial and non-financial interest is helpful to provide as part of this update. **C3**-Items are well considered, however a bit more clarification and definitions may be needed. This could be provided as examples too. **C4**-I have noted in one of my other answers that sometimes a funding agency is a non-profit or a professional society that receives funding from industry. An additional question is needed on this. **C5**-I have added a comment regarding one of the questions ( question 22) for your consideration. **C6**-**There is no item about preventing COI or how declarations of interests and evaluation of COI is factored into the formation of a guideline development group. This is crucial for an independent panel and also chair. I would strongly suggest including items that pertain to how COI factored into the selection of guideline committee members and chair and whether the chair was free of COI.** **C7**-Do not remember if covered in one of the items but it could be important to be explicit about the where does the funding is going (main aspects like salaries of methodologists/reviewers) and whether panellists and other guideline participants receive any funds. **C8**-Please consider the following:  1. Whether the declarations of interest were collected, ,reviewed, and reported to an independent body, or persons(s0 connected with the guideline) or guideline organisation).  2. Similar question for the management of COI.  3. Whether any person in the GDG has relationships (financial, commercial, scientific, academic, advisory, social, etc) with any industry (whether or not related to the content of the guideline).  4. Similar question about personal businesses. |  |
